# Silver CVD and ALD Precursors: Synthesis, Properties, and Application in Deposition Processes

**DOI:** 10.3390/molecules29235705

**Published:** 2024-12-03

**Authors:** Evgeniia S. Vikulova, Svetlana I. Dorovskikh, Tamara V. Basova, Aleksander A. Zheravin, Natalya B. Morozova

**Affiliations:** 1 Nikolaev Institute of Inorganic Chemistry, Siberian Branch, Russian Academy of Sciences, Ac. Lavrentiev Ave. 3, 630090 Novosibirsk, Russia; dorov@niic.nsc.ru (S.I.D.); basova@niic.nsc.ru (T.V.B.); mor@niic.nsc.ru (N.B.M.); 2 Meshalkin National Medical Research Center, Ministry of Public Health of the Russian Federation, Rechkunovskaya Str. 15, 630055 Novosibirsk, Russia; zheravin2010@yandex.ru

**Keywords:** silver, volatile precursors, structure, thermal properties, CVD, ALD

## Abstract

This review summarized the developments in the field of volatile silver complexes, which can serve as precursors in gas-transport reactions for the production of thin films and metal nanoparticles via chemical vapor deposition (CVD) and atomic layer deposition (ALD). Silver-based films and nanoparticles are widely used in various high-tech fields, including medicine. For effective use in CVD and ALD processes, the properties of silver precursors must be balanced in terms of volatility, thermal stability, and reactivity. In this review, we focus on the synthesis and comprehensive analysis of structural and thermal characteristics for the most promising classes of volatile silver complexes, as well as organometallic compounds. Following the specifics of silver chemistry, some features of the use of precursors and their selection, as well as several key directions to improving the efficiency of silver material deposition processes, are also discussed.

## 1. Introduction

Silver is a noble metal with truly unique properties. It has the lowest resistivity (1.59 µOhm·cm), the highest thermal conductivity, and a high reflection coefficient in the optical spectrum, making it attractive for the electronic industries [1,2]. In addition, silver demonstrates plasmonic and antibacterial effects, as well as chemical and (electro/photo)catalytic properties. These properties make it indispensable in modern high-tech fields such as biotechnology, sensors, and plasmon devices, as well as in the development of advanced catalytic systems [3,4,5]. In all of these fields, silver is most often used in the form of nanoparticles (AgNPs) or thin-film structures.

Like many thin metal coatings, silver films have a nanocrystalline structure [6]. In the early stages of their growth, they form separate agglomerates. Precise control over the morphology, thickness, and conformality of these layers is a key aspect in the process of creating materials with the necessary properties for each specific application. For example, ultrathin nanotextured coatings made of metallic silver are widely used in gas sensors, nanolithography [6,7], and catalysis [8]. Other technologically important applications of silver films include surface plasmon enhancement of fluorescence, luminescence, and Raman scattering. In addition, silver films are used in high-temperature superconducting systems, as absorbers for infrared sensors, and as waveguides [9,10,11,12]. 

It should be emphasized that, for plasmonic and optical devices, it is extremely important to have continuous thin silver films with extremely low surface roughness, whereas the thickness of the films varies from a few nanometers to several micrometers depending on the specific application [3]. On the other hand, when using silver layers as electrodes for organic solar cells [13] or to replace copper in integrated circuits [14], it is technologically important to reduce the width of the conductive lines. At the same time, the geometry of the structures covered can be extremely complex, including trench coats and other complex shapes. In recent years, silver nanostructures with high biocidal activity have been of particular interest. These structures can be introduced into the development and production of new-generation materials intended for biomedical technologies [15]. For such systems, as a rule, coated products with a non-planar geometry and/or a developed surface are used.

Currently, research in the field of silver nanoparticles (AgNPs) is attracting special attention, since nanoparticles have a larger surface-area-to-volume ratio than bulk metal, thus contributing to their high activity. The control of the size, shape, and, consequently, functional properties of AgNPs is crucial for their application in photovoltaics and sensors and as catalysts [16,17,18]. Due to surface plasmon resonance, AgNPs are used in functional optical coatings, sensors, biosensors, plasmon circuits, coherent light sources, and photovoltaic devices [17,19]. AgNPs play a key role in medicine and pharmaceuticals [17]. Due to their biocidal effect on various strains of microorganisms, they are widely used as antibacterial and antifungal agents [18,20,21,22].

Silver nanostructures/nanoparticles are usually obtained by three different methods [7,16]. The first approach is chemical. It consists in the reduction of metal ions in solution using various methods, such as wet chemistry, electrochemistry, sol–gel methods, and electrophoretic precipitation from aqueous suspensions [23]. One of the most common processes within the framework of the chemical approach is the reduction of AgNO_3_. This method makes it possible to obtain AgNPs both on the substrate surface and in the form of dispersed particles in colloidal systems, glasses, or polymers [15]. This approach is attractive due to its accessibility; however, it can lead to the inclusion of solution components in the composition of the material, which will negatively affect the functional properties. The use of a chemical approach is associated with the problem of recycling synthesis waste. In recent years, possible solutions to this problem based on the use of biological organisms for preparation of silvers nanomaterials have been proposed, but the number of publications in this area is still small [24,25]. 

The second method is physical vapor deposition (PVD), which includes metal evaporation, sputtering, or laser ablation [16,19]. In this case, resistive, electronic, and magnetron methods are traditionally used, but due to the ballistic trajectory of the sprayed substance, it is extremely difficult to ensure uniform deposition of the material on objects of complex shape, such as trench coats, porous structures, or nanofilaments [26]. The third approach can be considered a kind of combination of the first two, in which nanoparticles or films are formed as a result of a chemical reaction, but not from a solution, but from the gas phase of the precursor compound. This approach includes chemical vapor deposition (CVD), also known as metal–organic chemical vapor deposition (MOCVD), and atomic layer deposition (ALD), which are considered to be among the most precious and actively developing methods [27]. In recent decades, these methods have made significant progress due to the possibility of creating both simple (metallic and non-metallic) thin films and nanoparticles, as well as multicomponent structures under controlled conditions, as doing so is crucial for high-tech applications. These methods overcome the main disadvantage of PVD technologies: they are effective for covering objects of different shapes. In addition, they are usually characterized by simplicity of hardware design and the ability to scale [19]. Compared to “wet” chemistry, these methods make it possible to obtain cleaner materials.

In this review, we focus on the CVD and ALD processes for the production of silver films and nanoparticles since these methods represent a multifunctional tool for creating innovative film materials and nanostructures [16]. Since the main stage of deposition processes is a chemical reaction on the activated surface, the precursor plays a key role in these methods. However, to date, information on the main classes of volatile silver compounds suitable for such processes has not been summarized. Literature reviews on CVD and ALD of silver consider only a few key compounds or limited classes of complexes [28,29]. A detailed analysis of the chemistry and thermochemistry of volatile silver compounds can only be found in specialized reviews on carboxylates [30]. Therefore, in this review, we summarize information about volatile silver complexes for the first time, ranging from some features of their synthesis and structure to thermal properties and applications in CVD and ALD processes. 

## 2. Ag ALD and MOCVD: General Aspects and Requirements for Precursors

The starting compounds, known as precursors, play a key role in the ALD and MOCVD processes. High demands are placed on their properties, viz. sufficient volatility, thermal stability, the presence of a temperature window between vaporization and decomposition, and “clean” conversion to the target material without the formation of inorganic impurities. In addition, the properties of precursors, such as synthetic accessibility, storage stability, and non-toxicity, are important for practical use. However, the synthesis of silver compounds, which have all the necessary characteristics, is a difficult task due to the low charge density of the silver cation and its easy reducibility to metal, including under the influence of light. Compared with other noble metals, the Ag MOCVD and ALD processes are less deeply developed and difficult to implement [9]. This encourages researchers to look for new synthetic methods, classes of compounds, and approaches to organizing processes that can mitigate the requirements for precursors. It should be noted that the ALD and MOCVD methods have their own characteristics that affect the thermal and chemical properties of the precursors used.

**Figure 1:**
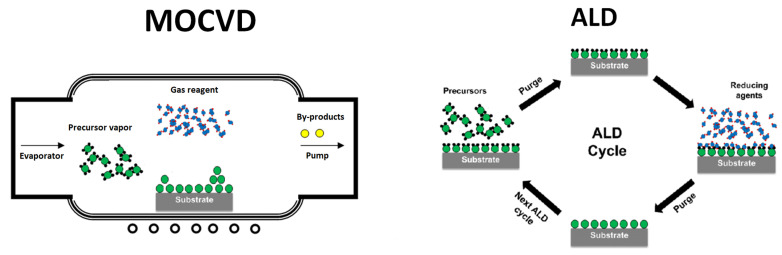
General schemes of MOCVD and ALD processes.

In general, the MOCVD process includes several sequential steps: (1) vaporization of an organometallic compound, (2) transportation of its vapors to the coated object, and (3) decomposition of the precursor and growth of inorganic material (Figure 1). 

In the classical version, vapors are transferred using an inert carrier gas, and the reaction on the substrate surface is thermally activated. The first experiment using silver was conducted in 1972 and was unusual: metal films were grown via the reaction of AgF vapors with a silicon substrate. Evaporation occurred directly at a pressure of 10^−6^ Torr [31].

After a long pause, work in this area intensified in the early 1990s, when metal–organic precursors began to be offered and tested. At the same time, alternative methods have been proposed to activate the decomposition reaction of the precursor, such as photo-irradiation [32,33] and even flame [34]. However, plasma-based processes known as PE-MOCVD have become the most widespread [35]. Due to the above-mentioned problems of silver compounds, alternative methods of introducing precursor vapors that do not require thermally stable and highly volatile complexes are actively being developed [9]. First of all, these methods include the use of direct liquid injection systems into the evaporator (DLI-MOCVD) [36] or the introduction of precursor solutions, for example, in the form of aerosol (aerosol-assisted, AA-MOCVD) [9,37,38] In this case, the solvent has a significant effect on the characteristics and composition of the resulting material. These approaches have significantly expanded the range of tasks that can be solved using Ag CVD methods.

At the same time, each modification of the MOCVD method has limitations in the deposition of highly conformal coatings or the conformal distribution of nanoparticles on complex three-dimensional surfaces, which are especially important for non-planar semiconductor and biomedical devices. In many applications of Ag-containing structures, control over the size, shape, and distribution of nanoscale elements on the surface is often crucial for their functional characteristics [7,39].

The ALD method, which is a chemical process for creating ultrathin films based on self-limiting reactions at the gas–solid interface, is especially effective for solving high-precision problems [26]. In this process, a metal-containing precursor reacts with surface groups on a substrate and, being in a chemisorbed state, interacts with a co-reagent (Figure 1). The repetition of these cycles leads to the formation of the necessary material [19]. In this method, alternating pulses of two or more gaseous reagents separated by purging with an inert gas are used. Under optimal conditions, the ALD process goes through saturation stages, leading to self-limiting film growth, providing maximum deposition control (up to the atomic level) [7]. Although initially such processes were developed to deposit thin continuous layers, today they are successfully adapted to the production of isolated nanoparticles [39].

It is important to emphasize that, in comparison with MOCVD, ALD processes impose more stringent requirements on the properties of precursors. Firstly, the precursors must have a high reactivity with both the functional surface groups of the substrate and the second co-reagent (reducing agent). Secondly, they must be thermally stable in the chemisorbed state before the introduction of the second co-reagent at the temperature of the ALD window. Finally, the precursors must be volatile in order to provide sufficient vapor concentration to saturate all surface centers. For this purpose, liquid precursors are usually used in evaporators. In such processes, as a rule, a one-time loading of precursors is performed to conduct the entire series of experiments. Therefore, long-term thermal stability at the sublimation/evaporation temperature is also important.

Ag ALD processes have been actively developing since 2007. In the few works devoted to this topic, two methods were mainly used: plasma-enhanced ALD (PE-ALD) and thermal ALD [7,19]. Plasma and other activations have several potential advantages, for example, lower reactor temperatures compared to thermal ALD. However, they also have their limitations, especially in the case of deposition of coatings on complex non-planar surfaces [7]. Precursor solution injection systems successfully used for MOCVD are also gradually adapting to ALD processes [40].

As is the case of most noble metals, at the initial stages of ALD, the process occurs through the formation of separate clusters or in the island mode of Vollmer–Weber growth, when metal atoms merge on a non-metallic surface [41]. This is probably due to the stronger interaction between silver atoms compared to the interaction between silver and the substrate [15]. The exact mechanism of the process is not fully understood, thus making it difficult to control the morphology on large surfaces of nanostructures. The type of precursor and the number of ALD cycles significantly affect the characteristics of the deposited material. The number of cycles determines the ability to control the processes of nucleation and growth of nanoparticles on the surface, which is especially important for structures consisting of dispersed particles of the same shape and size [23].

A common problem with silver thin films is their low chemical stability, since silver oxidizes in air and reacts with sulfur compounds in the atmosphere, leading to the formation of an Ag_2_S contaminant layer. This may complicate the use of silver in some areas [42]. Another problem of Ag ALD is low growth rates, which leads to low productivity and difficulties in scaling processes [41,42]. These problems can be solved by using PE-ALD at atmospheric pressure, i.e., APP-ALD. However, in this case, the requirements for the thermal properties of the precursor should be maximal: it is necessary to provide sufficient vapor pressure without using a vacuum, which requires relatively higher temperatures [42]. Another new activation method known as FEBID (Focused Electron Beam-Induced Decomposition) has recently been developed [43]. It can be used to create nanostructures by subjecting metal-containing precursors to electron-induced decomposition. However, unfortunately, the silver content in the obtained materials is often low.

Thus, the application of CVD and ALD methods opens the door to the creation of nanoscale materials with a diverse structure, surface morphology, physicochemical properties, and biological activity [15] on various types of 3D surfaces. These methods also allow the formation of dispersed AgNPs, and their shape and the size and degree of dispersion can be adjusted by optimizing the deposition parameters and choosing the type of chemical compound used as a precursor.

## 3. Silver Precursors for CVD and ALD Processes

In recent decades, CVD and ALD technologies have been actively developing, which requires the use of new silver precursors and optimization of deposition conditions. To ensure sufficient volatility, the precursors must be characterized by weak intermolecular interactions: the advantages of the precursors increase in the order of ionic << polymer << oligomeric < monomeric structure of the complex. In addition, the volatility of compounds can be further increased due to the presence of repulsive intermolecular contacts, such as F...F, which causes the popularity of fluorinated complexes [44].

However, due to the relatively large radius of the Ag^+^ ion (1.49 Å) [45]), the anionic ligands commonly used in volatile complexes (L = carboxylates, β-diketonates, amidinates, and pyrazolates) cannot completely saturate the coordination sphere of the metal center in the neutral fragment {AgL}. As a result, bridging bonds are formed, leading to the formation of poly- or oligomeric structures. Along with the high degree of ionization of silver bonds with donor atoms, this is the reason for the low volatility and low thermal stability of the compounds. A promising approach to solving this problem is the formation of adducts, i.e., “additional” coordination of neutral ligands [15,30,45] This makes it possible to saturate the coordination sphere of silver and obtain monomeric molecules or complexes with low nuclearity. However, in order to achieve this goal, it is necessary to ensure the strong binding of “additional” ligands. Since Ag^+^ is a “soft” metal center, it can be assumed that “soft” bases containing S-, P-, or N-donor atoms will be the most effective.

During the search for suitable precursors, a wide range of neutral ligands of various denticity were tested, including (cyclo)alkenes, alkynes, alkylisocyanides, (poly)amines, poly- and thioesters, derivatives of phosphines and phosphites, and dialkyl(di)sulfides. However, “soft” bases were not always the best choice [46,47]. For example, when using phosphines, in some cases, very stable complexes were formed, but the films obtained from them contained impurities of phosphorus and carbon, thus indicating incomplete removal of these ligands during decomposition [30]. On the other hand, the characteristics of the resulting complexes also depend on the anionic ligand. For example, cyclooctadiene-1,5 and its derivatives [48] proved to be effective for stabilizing low-nuclear fluorinated β-diketonate complexes, whereas there are no data on volatile carboxylate analogues.

Thus, when designing silver precursors, it is necessary to find the most suitable combinations of Q and L, or at least an acceptable balance between them. In this section, we briefly consider the key aspects of the synthesis, structure, and thermal properties of the obtained compounds, paying special attention to the classes of anionic ligand L.

### 3.1. Carboxylate Complexes

Silver carboxylates are one of the most actively studied substances for use in gas-phase processes. Earlier, scientists from Nicolaus Copernicus University collected achievements in this field and presented them in a thematic review on M(I) carboxylates [30]. Therefore, in this section, we will focus only on the most significant features of such complexes and provide information obtained from recent studies.

#### 3.1.1. Complexes Without Additional Ligand

**Figure 2:**
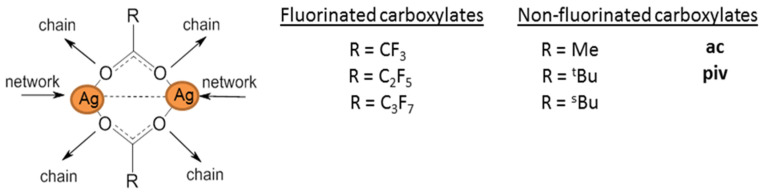
The general formulas of the main structural block in polymeric silver carboxylates and the ligands used to synthesize the volatile complexes.

Carboxylate ligands, L = COOR, are not chelating; however, they are in demand as ligands due to their low weight (respectively, to achieve a relatively high silver content in the complex) and the possibility of varying the properties of complexes by varying the R substituent. To ensure volatility, it is necessary to limit the molecular weight and dimensions of R. Acid derivatives containing no more than five carbon atoms in the main chain are usually used for this purpose (Figure 2).

These compounds are easily obtained as a result of exchange reactions between AgNO_3_ and carboxylic acid salts or directly by neutralization of Ag_2_O or Ag_2_CO_3_ with fluorinated carboxylic acid in aqueous alcohol solutions [30]. In the structures of complexes, due to bidentate syn–syn coordination of ligands, binuclear fragments {Ag_2_L_2_} are isolated (Figure 2). These fragments can be stabilized by an argentophilic contact (bond order 0.2, according to quantum chemical calculations [49]). Such building blocks form polymer networks (R = CF_3_ [49]) or chains (R = CH_3_ [50], *^t^*Bu [51]). At atmospheric pressure, all silver carboxylates decompose with the release of metal and carbon dioxide before or during melting, and the process temperature and volatile organic products are significantly dependent on R [11]. The temperature difference can be up to 250 °C, but, in general, non-fluorinated compounds are thermally less stable, which is associated with a lower covalence of the Ag-O bond [11].

At the same time, in vacuum, most of the complexes shown in Figure 2 are capable of sublimation (at least partially). Interestingly, a dynamic vacuum provides a higher yield than a static one [51]. This is due to the fact that, in a dynamic vacuum, vaporization begins at temperatures below the threshold of thermal decomposition. This effect was clearly demonstrated in a recent paper by J. Jurczyk et al. [52]. Fluorinated complexes are expected to be more volatile, whereas among the non-fluorinated ones, derivatives with bulk R are the most suitable. Mass spectrometric data show that fragments of {Ag_2_L_2_} are preserved in the gas phase [23], but the need to rupture polymer structures causes significantly higher sublimation temperatures than for mononuclear complexes with similar ligands.

In addition, the practical application of silver carboxylates is complicated by their high photosensitivity (storage problem) and low solubility, thus limiting their use in the AA-CVD method. However, it has recently been shown that the fluorinated complex (R = C_2_F_5_), which forms an adduct with water [Ag_5_(L)_5_(H_2_O)_3_]_n_, demonstrates better stability in air and light compared to the initial [Ag(L)]_n_ [23]. This is probably due to a decrease in the number/strength of argentophilic interactions that can contribute to the formation of metallic silver [50]. Taking into account the dehydration stage, the mass spectrum of the vapor of this adduct and its change with temperature are typical for silver carboxylates. Thus, the formation of such adducts can contribute to improving the storage characteristics of complexes, making them more suitable for practical use.

Another interesting approach, which has recently been proposed, is the formation of carboxylates in situ as a result of thermal transformations of ionic precursors. Such precursors can be more stable during storage. In particular, a series of complexes [Ag(NH_2_*^t^*Bu)_2_](L)⋅xH_2_O (R = CF_3_, C_2_F_5_, and others) were synthesized by direct reaction of [Ag(L)]_n_ with tert-butylamine in toluene with high yields [18]. Upon decomposition of complexes with small R substituents under reduced pressure, amine cleavage occurs with the formation of volatile fragments such as {AgL} and {Ag_2_L_2_}. Although the quality of the films obtained using this method does not yet meet expectations [18], the ability to control such transformations is of practical interest.

#### 3.1.2. Silver Carboxylate Adducts

Silver carboxylate adducts began to be actively studied at the early stages of the development of volatile silver-containing precursors. Lewis P-donor bases were mainly used as neutral Q ligands. The main method of synthesis was the direct interaction of Q with carboxylate Ag(L) in an inert atmosphere.

*Adducts with tertiary phosphines.* Most of the work in this area was described in detail in the above-mentioned review published in 2005 [30]. A wide range of carboxylates was tested (subsequently L = dimethylbutyrate [53] was added to them), and Q = PMe_3_, PEt_3_, PnBu_3_, and PPh_3_ were mainly used as neutral ligands. In brief, mononuclear derivatives [Ag(Q)_2_(L)] or [Ag(Q)_3_(L)], as well as stoichiometry complexes [Ag(Q)(L)]_n_ having dimeric, tetrameric or polymer structures can be formed in the synthesis processes. Mononuclear complexes are characteristic of the combination of a bulk phosphine with a relatively small carboxylate ligand, whereas with an increase in the volume of the latter (*^t^*Bu or C_2_F_5_ substituent), two or three such phosphines (P*^n^*Bu_3_, PPh_3_) can no longer enter the coordination sphere of a single silver atom, thus leading to a change in stoichiometry and dimerization. The use of small-sized phosphines such as PMe_3_ leads to the formation of polymer chain structures, for example, in [Ag(OOCC_2_F_5_)(PMe_3_)] [54].

The differences in the structure of these compounds are manifested in their thermal properties. According to mass spectrometric data, for complexes with polymer structures and/or having short Ag...Ag contacts, decomposition begins with the cleavage of a neutral ligand, for example, PMe_3_. The ease of cleavage of the Lewis base also correlates with a decrease in its σ-electron donation ability [53]. For derivatives of other phosphines, on the contrary, a carboxylate fragment cleaves off at the same temperatures, forming volatile silver–phosphine species. This affects the impurity composition of the films (see Section 4). Depending on the temperature, the decomposition pathway may vary, but, in general, the stability of the Ag-O bond in the case of perfluorinated carboxylates is lower. However, as expected, an increase in the mass of phosphine leads to a decrease in the volatility of the complexes.

In general, the above-mentioned review published in 2005 [30] noted the non-fluorinated derivatives with Q = P^n^Bu_3_ as promising for classical CVD, probably as a compromise option. Subsequently, it was this phosphine that was used to expand a number of carboxylate adducts, starting with the simplest representatives of this class (L = OOCCH_3_ and OOCCF_3_) and ending with aromatic and dicarboxylic derivatives (squarate, oxalate, and their homologues, including unsaturated ones [54,55,56]. However, the structure of these complexes has not been confirmed by the XRD method, and the thermal properties are presented in a fragmentary form, which does not allow us to identify any regularities, despite the fact that a large number of compounds (about 20) have been obtained. As a result, only two adducts were selected by the authors for CVD testing: [Ag(Q)_2_(OOCCF_3_)] [56] and [Ag(Q)_3_(O_2_CCH_2_CO_2_)] [55]. 

It should be noted that the work [56] also described the synthesis of the corresponding adducts with *phosphites* P(OMe)_3_ and P(OEt)_3_; however, no discussion or primary data for them were given.

*Adducts with diphosphines* were also described in detail in the same review [30]. Such neutral ligands affect the number of metal centers in the complex and illustrate the variability of the ways in which carboxylic acid ions coordinate, forming di-, quad-core or polymer structures. Thus, despite the rich structural diversity, within the framework of this concept, it has not yet been possible to obtain mononuclear adducts or compounds with thermal properties that would be promising for the considered application.

**Figure 3:**
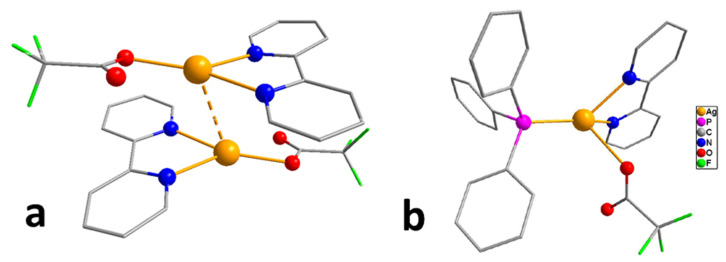
Silver carboxylate adducts with 2,2-bipiridine: binuclear [Ag(bipy)(OOCCF_3_)]_2_ (**a**); mononuclear [Ag(PPh_3_)(bipy)(OOCCF_3_)]_2_ (**b**). Hydrogen atoms were omitted for clarity. The cif-files were retrieved from CCDC. CCDC numbers are 734740 and 602583, respectively.

*Adducts with chelating neutral ligands*. 2,2-bipyridine (Q = bipy) was used as a chelating neutral ligand in combination with perfluorinated carboxylates (L = OOCR^F^, R^F^ = CF_3_, C_2_F_5_, C_3_F_7_) [10]. Interestingly, all the obtained complexes had different stoichiometry Q:AgL. In the case of more voluminous L, chain structures, where the neutral ligand also served as a bridge, were formed (Q:AgL = 1:2 when R^F^ = C_2_F_5_ or 4:6 when R^F^ = C_3_F_7_). When R^F^ = CF_3_, the complex had a stoichiometry Q:AgL = 1:1, but it was a dimer with a shortened contact Ag...Ag, so that the carboxylate ligand remained monodentate-bound (Figure 3a). Polymer complexes turned out to be more thermally stable than dimeric ones (the difference in threshold temperatures was about 50–60 °C). Direct sublimation tests were not performed, but both molecular peaks and [Ag(Q)]^+^ or metal-containing products of its defragmentation were absent in the mass spectra. At the same time, {Ag_2_L_2_} fragments characteristic of carboxylates were observed, and the intensity of [Q]^+^ was also high, which could indicate the cleavage of the neutral ligand during vaporization. Unfortunately, attempts to obtain Ag coatings using the classical version of the MOCVD method proved unsuccessful.

Thus, among the many carboxylate adducts, only a few derivatives of tertiary phosphines turned out to be suitable for the MOCVD method. However, in order to obtain a clean product without impurities, it is necessary to optimize the deposition conditions (see Section 4). At the same time, the low thermal stability of these derivatives significantly limits their use in ALD. In addition, a series of adducts combining both types of neutral ligands have been synthesized, such as [Ag(PX_3_)(bipy)(L)], which have mononuclear molecular structures (Figure 3b) [57]; however, the information about their thermal properties is not available.

### 3.2. β-Diketonate Complexes

This class of compounds is one of the most demanded in MOCVD technologies, and this demand is explained by the availability and stability of such complexes [28]. At the same time, the relatively low reactivity of metal β-diketonates limits their use in ALD methods. Nevertheless, in the case of noble metals, they are actively used in ALD processes based on reduction reactions [58]. Although there are many thematic reviews devoted to volatile β-diketonate metal complexes in the literature [28,59,60], so far, silver has not been the subject of detailed discussion.

#### 3.2.1. Complexes Without Additional Ligands

**Figure 4:**
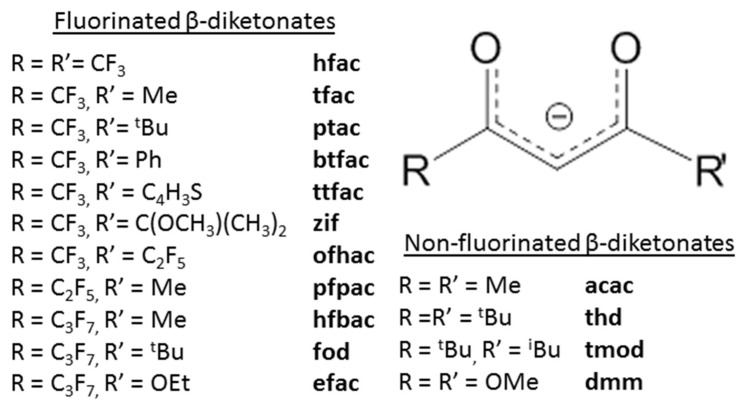
The general formula abbreviation of the main β-diketonate ligands used to synthesize silver complexes.

Although β-diketonate ligands (L = RC(O)CHC(O)R′) have a higher weight and carbon content than carboxylate ligands, they favorably differ in the presence of a chelate effect and conjugation in the organic part of the metallocycle, which stabilizes the complexes. In addition, the possibility of introducing two different substituents R and R′ increases the variety of properties of the complexes. The introduction of fluorine atoms into these terminal groups and an increase in the volume of substituents usually leads to an increase in the volatility of the complexes due to intermolecular repulsion or loosening of the crystal packaging [58]. However, there are limitations on increasing the volume of substituents. As in the case of carboxylates, ligands with substituents R and R′ containing no more than five carbon atoms in the backbone are usually used for MOCVD/ALD applications, since with a further increase in the size of the substituent, the volatility of the complexes decreases significantly due to an increase in molecular weight. Figure 4 shows the β-diketonate ligands proposed for the synthesis of silver complexes.

Two main methods are used to synthesize these compounds, viz. the acid–base interaction of Ag_2_O with fluorinated β-diketone HL_F_ and the exchange reaction of AgNO_3_ with weaker HL acids (non-fluorinated derivatives are pre-deprotonated with triethylamine). Traditionally, reactions are carried out in a Schlenk line [61,62]; however, for some fluorinated complexes, it was found that inert conditions are not always required [63,64]. During synthesis, the reaction mixture must be cooled and protected from light, especially for non-fluorinated derivatives, which are particularly sensitive to external influences.

Due to the coordination unsaturation of the metal center in the {Ag(L)} fragment, all known β-diketonates of silver have polymer structures. These complexes often include molecules of water or solvents such as CH_3_CN, THF, or toluene, which expands their structural diversity but does not decrease the dimension of the structure [61,62,63,64]. In this aspect, a bulk substituent has a positive effect: in the case of L = tfac (R = CF_3_, R′ = CH_3_), a network structure is formed (including Ag...Ag contacts), whereas an increase in both the fluorinated (R = C_2_F_5_) and non-fluorinated (R′ = *^t^*Bu) groups leads to the formation of chain polymers [64,65].

It is important to note that, unlike carboxylates, whose structures are based on {Ag_2_(L)_2_} fragments, it is impossible to isolate a common building block for silver β-diketonates. This suggests that there are no stable particles in these compounds that could pass into the gas phase when the polymer structure is destroyed. Apparently, this is the main reason for the decomposition of β-diketonates of silver when heated, and even in vacuum conditions, vaporization is not observed. Because of this, such compounds are not suitable for classical MOCVD, although their high solubility in typical organic solvents (especially for ligands with an increased perfluorinated group) [64] may be of interest for alternative precursor entry systems.

#### 3.2.2. Beta-Diketonate Adducts

Since silver β-diketonates themselves, unlike carboxylates, turned out to be unsuitable for most MOCVD processes, research into the development of their adducts was carried out more actively. To stabilize low-nuclear complexes, a wide range of neutral ligands of various denticity and with different donor centers, which are shown in Table 1, were tested. On the other hand, hexafluoroacetylacetonate (L = hfac; R = R′ = CF_3_) was considered as an anionic ligand in most studies. The predominant methods of adduct synthesis are the reaction of the corresponding β-diketonate Ag(L) with a neutral ligand (Q) or in situ assembly from Ag_2_O, Q, and β-diketone HL in an organic solvent under inert conditions (Schlenk technique). Other methods will be considered below. Most complexes are photosensitive, so they should be stored in light-tight vessels at low temperatures.

*Adducts with π-donor ligands*. Since silver cycloolefin compounds with β-diketonates Table 1 have been known since the 1970s and are easily obtained from AgNO_3_, a neutral ligand, KL, or NaL in air in an aqueous solution [66], this type of adduct was among the first whose structure and properties were studied for possible use in MOCVD. Vinyltriethylsilane (VTES) and alkyne derivatives, including bis-(trimethylsilyl)acetylene (BTMSA), were tested among acyclic ligands [67,68,69].

**Figure 5:**
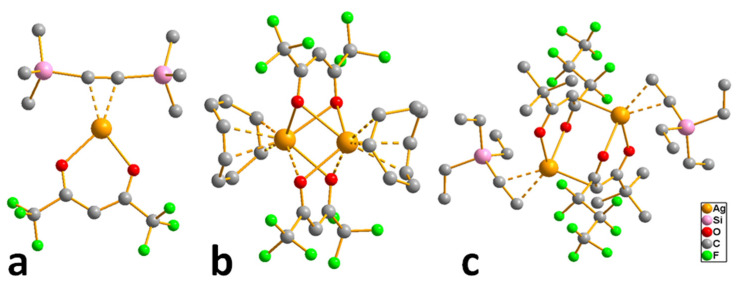
The molecular structures of silver β-diketonate adducts with π-donor ligands: [Ag(BTMSA)(hfac)] (**a**); [Ag(cod)(hfac)]_2_ (**b**); [Ag(VTES)(fod)]_2_ (**c**). Hydrogen atoms were omitted for clarity. The cif-files were retrieved from CCDC. CCDC numbers are 212251, 1173700, and 1271428, respectively.

Most of the obtained complexes have stoichiometry Q:AgL = 1:1 and are characterized by moderate stability during storage. However, compounds with olefins, with the exception of cyclooctadiene-1,5 (cod) and norbornadiene (nbd) derivatives [70,71,72], quickly lose their neutral ligand in air. Moreover, a decrease in the degree of fluorination of L significantly destabilizes these complexes [70]. Interestingly, such regularity was not observed for acyclic BTMSA and VTES [68,69]. It is important that, in the case of VTES, it was possible to obtain three liquid precursors (L = hfac, tfac, and ofhac with R = CF_3_ and R′ = CF_3_, CH_3_, and C_2_F_5_, respectively) [68,71], whereas the rest of the compounds were crystalline. All alkyne derivatives have a mononuclear structure (Figure 5a), whereas the known structures of complexes with cod and VTES are binuclear (Figure 5b,c). At the same time, the structural organization of dimers differs: fragments of Ag(VTES)(L) are combined due to paired bonds of Ag-C with the methyl group L [68], whereas in the case of cod, oxygen atoms perform the bridging function [71,73]. Complexes of the second type melt at higher temperatures (~80–120 °C) and, accordingly, are characterized by greater thermal stability, since melting is incongruent for all compounds of this type.

Liquid Ag(VTES)(L) complexes with perfluorinated ligands (L = hfac, ofhac) demonstrate a partial transition to the gas phase already at atmospheric pressure, which is confirmed by TGA [71]. Solid adducts are characterized by cleavage of a neutral ligand. Of the whole variety, only Ag(BTMSA)(L) (L = hfac and fod with R = C_3_F_7_ and R′ = ^t^Bu) were sublimated under relatively mild conditions (30–40 °C, 0.1 Torr) [69]. However, both complexes have limited thermal stability: they melt incongruently at 64 and 88 ° C, respectively.

In this regard, the most commonly used precursor in this class was [Ag(cod)(hfac)]_2_, which is much less volatile but more thermally stable (up to 120 °C). In order to ensure sufficient mass transfer, it is necessary to create special conditions for vaporization (see Section 4.1). The volatility assessment of [Ag(cod)(hfac)]_2_, which is important for optimizing this process, was carried out by Zherikova et al. [74]. In addition, it has recently been found that a small increase in the perfluoroalkyl chain of β-diketonate (L = ofhac) may slightly increase the volatility of [Ag(cod)(L)]_2_ without loss of stability [71]. 

*Adducts with P-donor ligands*. As classical representatives of “soft” bases, P-donor ligands were selected to stabilize silver β-diketonates (Table 1), similar to the case of carboxylates described above (see Section 3.1.2). Various tertiary phosphines (Q = PMe_3_, PEt_3_, PnBu_3_, PPh_3_) and phosphites (Q = P(OMe)_3_, P(OEt)_3_, and P(O*^i^*Pr)_3_ were tested [12,75]. The corresponding complexes [Ag(Q)(L)] are easily synthesized with high yields both using traditional methods (described earlier) and by substituting an alkene in the corresponding adduct [76]. With initial stoichiometry AgL:Q = 1:2, the adducts of the corresponding composition [Ag(Q)_2_(L)] can be obtained, as was shown for both small Q = PMe_3_ [77] and sterically hindered Q = PPh_3_ [78]. The only exception is the combination of the most voluminous phosphine with β-diketonate: for Q = PPh_3_ и L = fod, the yield decreased to 30%, and the product had an unexpected stoichiometry of AgL:Q = 3:5 [79]. It is important to note that the use of phosphines made it possible to stabilize a number of non-fluorinated silver β-diketonates (Table 1) [61,79], and this has not been achieved for any other type of neutral ligands. Compared with π-donor adducts, complexes of the current class are characterized by significantly greater light stability.

**Figure 6:**
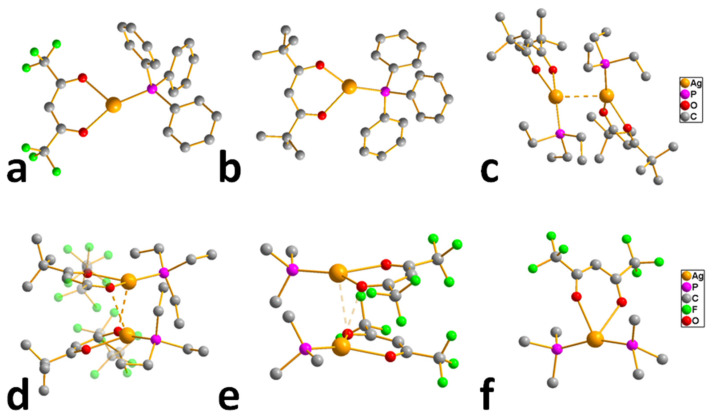
The molecular structures of silver β-diketonate adducts with phosphines: [Ag(PPh_3_)(hfac)] (**a**); [Ag(PPh_3_)(thd)] (**b**); [Ag(PEt_3_)(thd)]_2_ (**c**); [Ag(PEt_3_)(fod)]_2_ (**d**); [Ag(PMe_3_)(hfac)] (**e**); [Ag(PMe_3_)_2_(hfac)] (**f**). Hydrogen atoms were omitted for clarity. The cif-files were retrieved from CCDC. CCDC numbers are 152580, 169487, 809279, 809280, 1204823, and 1204824, respectively.

Another interesting feature is that complexes with Q = PEt_3_ have extremely low melting points: 45–47 °C and 25–28 °C for L = hfac and fod, respectively. Most complexes with PEt_3_ and P^n^Bu_3_ and all phosphite adducts are liquid. The crystal structure has been determined for only a few adducts (Figure 6). The AgL:Q = 1:2 stoichiometry provides a mononuclear molecular structure (Figure 6f), whereas, in the case of AgL:Q = 1:1, variations are possible depending on the combination of Q and L. In particular, the most voluminous phosphine, Q = PPh_3_, supports a monomeric structures (Figure 6a,b), while the diminishment of substituents in phosphine to ethyl ones already allows the formation of binuclear complexes (Figure 6c,d). The structural organization of the latter is determined by the β-diketonate ligand: this can be bonded via argentophilic contacts (non-fluorinated L = thd, Figure 6c) or via elongated Ag-O bonds (fluorinated bulk L = fod, Figure 6d). Note that the last motif can also be traced in the structure of the fluorinated adduct with the smallest Q = PMe_3_ (Figure 6e); however, the distance here is significantly greater (2.97 vs. 2.68 Å), so that [Ag(PMe_3_)(hfac)] still has a mononuclear structure. In general, such relatively weak joining contacts are not expected to prevent the transition to the gas phase in monomeric form. However, the presence of argentophilic interactions may reduce the thermal stability of non-fluorinated adducts by favoring the formation of metallic silver.

The thermal stability of adducts decreases during the transition from fluorinated β-diketonates (L = hfac > tfac ~ fod) to non-fluorinated ones [79]. However, the derivative with Q = PBu_3_, L = thd, (R = R′ = ^t^Bu), apparently, is the transition to the gas phase (TGA data) [61]. This is the only known example of a volatile non-fluorinated β-diketonate silver complex. Almost all fluorinated complexes are easily distilled or sublimated. As expected, an increase in volume of substituents in phosphine led to a decrease in volatility [76]. Molecular peaks were recorded in the mass spectra of phosphite derivatives [12]. However, the processes of thermal decomposition of these complexes have not been studied in detail. Since phosphorus impurities are usually not detected in films obtained from these compounds [79], it can be assumed that their destruction occurs by a different mechanism than that of carboxylates, which we discussed earlier: Ag–phosphine fragments are not formed in the gas phase. This type of decomposition, in which the anionic ligand is cleaved off first, makes these compounds widely used in ALD processes.

*Adducts with S-donor ligands*. Based on the successful experience with phosphines, it could be assumed that S-donor ligands would be able to effectively stabilize the complexes, since they are also “soft” Lewis bases. Indeed, the adducts of [Ag(Q)(hfac)] were obtained with high yields for 1,4-oxathiane (C_4_H_8_OS) and a series of Q = SMe_2_, SEt_2_, SnPr_2_, and SnBu_2_ (Table 1) [80]. It is noteworthy that the last two compounds turned out to be liquid. However, when attempting sublimation, decomposition was observed even at reduced pressure (0.05 Torr), and the condensate composition did not correspond to the original compound.

*Adducts with O-donor ligands*. Like classical “hard” Lewis bases, ligands of this type have proved ineffective for stabilizing silver complexes. In particular, polyesters CH_3_O(CH_2_CH_2_O)mCH_3_ (m = 2 (diglyme), 3 (triglyme), 4 (tetraglyme)) were tested for L = hfac (Table 1) [36,81]. The obtained complexes had dimeric (similar to [Ag(cod)(hfac)]_2_, Figure 5b), ionic ([Ag(Q)_2_][Ag(hfac)_2_]) and monomeric structure, respectively. Although the molecular complexes turned out to be low-melting, all adducts decomposed at both atmospheric and low pressure.

**Figure 7:**
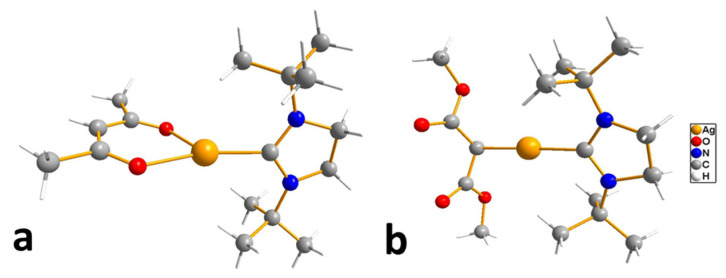
The molecular structures of silver β-diketonate adducts with N-heterocyclic carbene: [Ag(*^t^*^Bu^NHC)(acac)] (**a**); [Ag(*^t^*^Bu^NHC)(dmm)] (**b**). The cif-files were retrieved from CCDC. CCDC numbers are 2104342 and 2104340, respectively.

*Carbene adducts*. To increase the reactivity of adducts, it has recently been proposed to use “additional” ligands that ensure the formation of organosilver fragments [41]. N-heterocyclic carbenes (NHCs) are effective as such ligands, in which bulk alkyl/aromatic substituents provide the necessary electron-donating and steric characteristics [82]. Keeping in mind the volatility, i.e., restrictions for substituents similar to those described above for β-diketonates, 1,3-di-tert-butyl-imidazolin-2-ylidene (*^t^*^Bu^NHC) was chosen to stabilize Ag(I) complexes [83]. The synthesis of the corresponding adducts (Table 1) was carried out from a silanaminide precursor (see Section 3.3.3) by direct substitution of the anionic part with β-diketone, and the yields of fluorinated derivatives were significantly higher than those of non-fluorinated ones [83]. All compounds had a mononuclear structure, and the bulk *^t^*^Bu^NHC fragment effectively shielded the metal center, preventing additional argentophilic contacts. It is noted that when electron-donating substituents are introduced into the β-diketonate ligand, its classical chelate coordination (L = hfac, fod, and acac) gives way to binding due to the methine carbon atom (L = dmm and dimethylmalonate) (Figure 7). The thermal stability of the adducts decreases in the following order: L = fod > hfac > acac > dmm (DSC data). Non-fluorinated compounds are non-volatile, and both the low-intensity peaks of molecular ions and high-intensity [Ag(NHC)]^+^ are recorded in the mass spectra of fluorinated adducts. The latter indicates that the Ag-carbene bond is stronger than the Ag-L one (at least under electron impact). This type of decomposition is promising for the implementation of ALD processes (see Section 4.2).

*Adducts with N-donor ligands*. Unlike carboxylates, where the choice of such ligands was limited to Q = bipy, a wider range of derivatives were tested to prepare β-diketonate-adducts, including aliphatic di- and poly(amines), aromatic bidentate heterocycles, and a scorpionate-type-molecule Table 1. Basically, adducts were synthesized for L = hfac [36,84,85]. Only in recent studies, using Q = bipy and HC(3,5-Me2pz)_3_ (tris(3,5-dimethylpyrazole-1-yl)methane), researchers began to vary the β-diketonate ligand (Table 1) [47,86].

Along with the “classical” synthesis method (see above), a method of in situ self-assembly of AgNO_3_, Q, and NaL in an aqueous solution was described for Q = bipy [84]. However, it has recently been discovered that this reaction is complicated by the formation of cationic complexes [Ag(bipy)_2_]^+^ [86]. A similar competitive coordination was observed for scorpionate Q = HC(3,5-Me_2_pz)_3_, but this system is significantly more labile [47]. Tetradentate polyamine can also saturate the coordination sphere of silver, as shown using an ionic complex [Ag(hmten)][Ag(hfac)_2_], as an example, but its formation is clearly regulated by the stoichiometry of synthesis [85].

**Figure 8:**
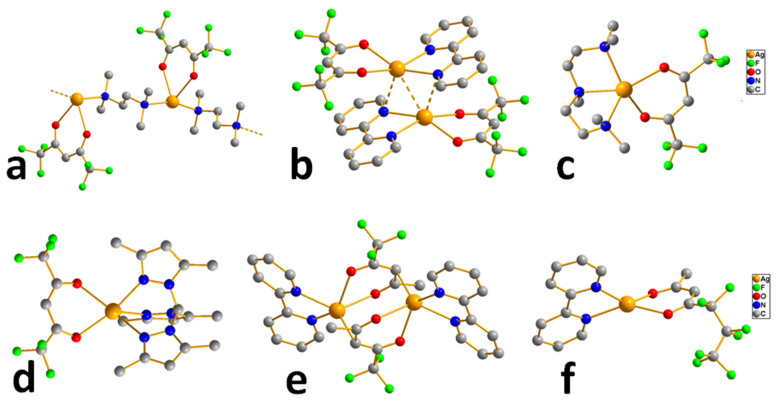
The molecular structures of silver β-diketonate adducts with N-donor ligands: [Ag(tmeda)(hfac)]_∞_ (**a**); [Ag(bipy)(hfac)]_2_ (**b**); [Ag(pmdien)(hfac)] (**c**); [Ag(HC(3,5-Me_2_pz)_3_)(hfac)] (**d**); [Ag(bipy)(tfac)]_2_ (**e**); [Ag(bipy)(hfbac)] (**f**). Hydrogen atoms were omitted for clarity. The cif-files were retrieved from CCDC. CCDC numbers are 209037, 209036, 1220656, 2366526, 2309420, and 2309419, respectively.

Despite the described difficulties, it was possible to synthesize adducts with stoichiometry Ag(L):Q = 1:1 for all studied combinations of Q and L. Crystal structures have been determined for most of them. At a fixed L = hfac, the effect of the neutral ligand Q on the structure of the complex can be traced. In the case of sterically labile bidentate Q = tmeda (N,N,N′,N′-tetramethylethylenediamine), a chain polymer was formed due to the bridging function of the amine (Figure 8a) [84]. For aromatic Q = bipy and phen (1,10-phenanthroline), dimers were formed due to Ag...Ag contacts (Figure 8b) [36,84]. An increase in the denticity of ligands makes it possible to stabilize mononuclear molecules (Figure 8c,d) [47,85]. Another effective strategy is the modification of the β-diketonate ligand, which was demonstrated by the example of Q = bipy. The introduction of an unfluorinated group changes the structural organization of the dimer (Ag-C bonds appear), and an increase in the fluorinated group leads to the formation of a mononuclear complex (Figure 8e,f) [86].

When heated under atmospheric pressure, all adducts undergo decomposition (TGA data); however, polyamine derivatives can be quantitatively sublimated at temperatures from 80 to 100 °C and a pressure of 0.01 Torr. When using the same L = hfac, complexes with Q = bipy and tmeda are also able to transition to the gas phase, but at a lower vacuum of 0.001 Torr, which correlates with the greater nuclearity of the structures (and additional stacking in the case of Q = bipy). 

Thus, the use of N-donors opens up new horizons for the creation of volatile β-diketonate complexes of silver. However, additional research is needed to find the optimal combinations of neutral and anionic ligands.

### 3.3. Complexes with N-Donor Anionic Ligands

N-donor anionic ligands are also widely used to create volatile complexes, since they have two important advantages. On the one hand, they can favor the production of pure metal (without admixture of oxide phases due to the absence of a direct M-O bond, which is important for MOCVD). On the other hand, they provide increased reactivity (due to presence of M-N bonds), which is especially important for efficient ALD processes.

#### 3.3.1. Amidinate, Guanidinate, and Related Complexes

Active research on compounds of these classes for use in CVD and ALD began in the mid-2000s [87,88]. Among the metals of group 11, only copper complexes were mainly studied [89]; therefore, by now a fairly small set of corresponding silver derivatives has been studied.

**Figure 9:**
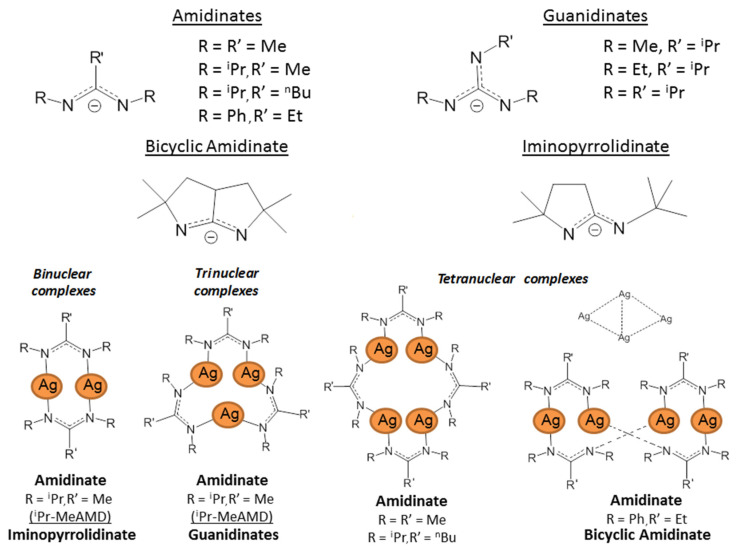
The main types of silver complexes with N-donor anionic ligands.

Amidinate ligands (L = R_2_N_2_CR′, Figure 9) can be considered derivatives of carboxylates (O_2_CR′), which have a large structural diversity due to the “additional” ability to vary R substituents at donor nitrogen atoms. 

The further introduction of the N(R′)_2_ group at the methine carbon atom, i.e., the transition to guanidinate ligands (L = R_2_N_2_CN(R′)_2_, Figure 9), adds another center to the conjugated π system, which may contribute to an increase in the stability of the complexes. Another method of stabilization consists in the inclusion of one or both donor atoms in a cyclic fragment, that is, in the transition to iminopyrrolidinates or bicyclic derivatives (Figure 9). Amidinates and related classes are readily available and, as a rule, do not contain fluorine, which is important for a number of applications. Figure 9 shows the structures of the ligands proposed for the preparation of silver complexes. As for the anions discussed above, substituents no larger than ^t^Bu are mainly used in this case.

The synthesis of complexes is carried out in an inert atmosphere with protection from light exposure. Classical methods are based on the reaction of AgCl with the corresponding carbodiimide or iminopyrrolidone deprotonated with butyllithium [90,91]. To obtain guanidinates, ^n^BuLi is replaced with the corresponding dialkylamide [92]. In the case of a bicyclic derivative, the use of potassium bis(trimethylsilyl)amide and silver acetate is already required [93]. Although the yields for derivatives with cyclic fragments turned out to be noticeably lower, these complexes are attractive for their long-term stability when stored in air.

In crystals and in solution, all guanididates have a trinuclear structure, iminopyrrolidinate is binuclear, and bicyclic amidinate is tetra-nuclear (Figure 9). Depending on the combination of substituents, amidinate complexes can form all types of structural ensembles. Tetra-nuclear complexes additionally differ in spatial organization (cyclic substituents bring metal centers closer together; Figure 9). Interestingly, for the simplest amidinate (R = ^i^Pr and R′ = Me), a dimer–trimer equilibrium is observed in solution, while the solid phase is a co-crystallization of both of these forms [90]. All structures are stabilized by argentophilic contacts, and according to quantum chemical calculations, this interaction is about twice as strong in the dimer [91]. Therefore, similar to silver carboxylates, it is possible to expect stabilization of fragments {Ag_2_(L)_2_} in the gas phase, which is more favorable for the vaporization of binuclear complexes.

In fact, trinuclear guanidinates decompose during sublimation [92], whereas the binuclear amidinate and iminopyrrolidinate, on the contrary, showed attractive thermal characteristics. The first complex sublimes quantitatively in vacuum (80 °C, 0.04 Torr) [90], and the second one is capable of evaporation already at atmospheric pressure (isothermal TGA data at 180–210 °C) [94]. According to the DSC, iminopyrrolidinate remains stable up to 290 °C. Unexpectedly, the tetra-nuclear complex (bicyclic amidinate) also demonstrates the ability to sublimate, although it is significantly less volatile compared to the binuclear related complexes (160–170 °C and 0.03 Torr) [93]. This can be explained by the reversible dissociation into two fragments {Ag_2_(L)_2_} during vaporization, which is facilitated by the structural organization of the {Ag_4_(L)_4_} ensemble of this type (characterized by the close arrangement of silver atoms, Figure 9).

Thus, promising volatile non-fluorinated silver precursors can be obtained on the basis of amidinate ligands without formation of adducts. Cyclic derivatives of these compounds are characterized by increased thermal stability. This is consistent with the fact that the most favorable thermolysis pathways of such complexes include elimination of carbodiimide (in solutions, low temperature) or β-hydrogen (for vapors, high temperature) [89]. However, the drawback of such complexes is relatively high melting points.

#### 3.3.2. Pyrazolate Complexes

Pyrazole-based ligands are common building blocks in coordination chemistry. They are used to synthesize bioactive compounds, catalytic structures, organometallic frameworks, etc. [95,96,97]. However, their use in the development of volatile complexes is still limited. In particular, only two papers considering the corresponding silver derivatives as potential MOCVD/ALD precursors have been found [19,46].

**Figure 10:**
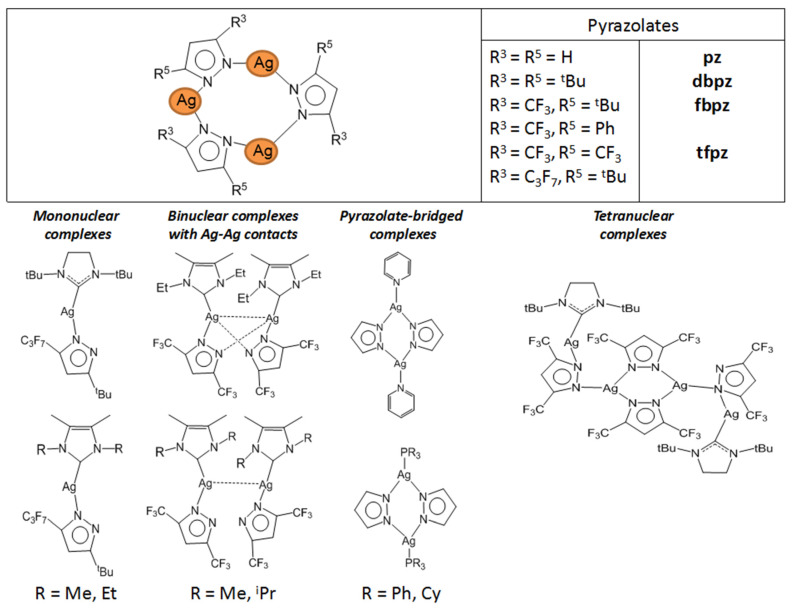
Silver pyrazolate complexes.

*Complexes without additional ligand.* Since pyrazolate ligands (Figure 10) have neighboring donor atoms in a heterocycle, they can be expected to have a bridging function, rather than coordination with a single metal center. At the same time, the structure of the complexes can be changed by combining two substituents in three and five positions (HL = 3-(R),5-(R′)PzH) and/or adduction. Silver pyrazolates are readily available compounds: the entire set of these complexes containing substituents suitable for volatile precursors (see above) can be obtained by the reaction of Ag_2_O and HL under inert conditions [46,98]. These complexes have a three-nuclear structure (Figure 10), and their crystal packing is stabilized by intermolecular Ag···Ag contacts, which have a length comparable to van der Waals radii. This is reflected in the relatively high melting temperatures (120–190 °C), even for fluorinated derivatives.

To test the thermal properties, three silver pyrazolates with substituents of different natures were selected (R and/or R′ = CF_3_, *^t^*Bu, Figure 10) [46]. The non-fluorinated complex decomposed upon heating, whereas the fluorinated analogues (R = CF_3_) already showed noticeable volatility at atmospheric pressure, which naturally increases in the order R′ = *^t^*Bu < CF_3_. Mass spectrometric data confirmed that the three-nuclear structure was preserved in the vapor phase. It was noted that both complexes can be sublimated in a low vacuum (110–135 °C, 0.5 Torr), but their volatility has not been compared with other classes of silver precursors. Nevertheless, testing in the MOCVD experiments yielded interesting results [46].

*Pyrazolate adduct.* In order to reduce the nuclearity of the structure of silver pyrazolates to increase the reactivity for ALD processes, two types of monodentate neutral ligands were tested: N-donor (pyridine, Py) and N-heterocyclic carbenes (NHCs, Figure 10). Only fluorinated derivatives were considered as anionic ligands. 

*The adduct with pyridine* was obtained with good yield by in situ assembly reaction from Ag_2_O, Py and Htfpz (R = R′ = CF_3_) [46]. Presumably, it has a binuclear structure due to the bridging function of both pyrazolate ligands (Figure 10). However, the binding of the neutral ligand to the metallocenter turned out to be insufficiently effective: when heated, it cleaved off and a three-nuclear pyrazolate was formed. It can be assumed that phosphine adducts is more stable, but their thermal properties have not been studied [99].

*Carbene adducts* have been synthesized by direct reaction of a neutral ligand and silver pyrazolate [19]. A wide range of complexes have been obtained, and it was shown that the yields and the structure of adducts depend on both neutral and anionic ligands (Figure 10). It is noteworthy that the structure of the donor node and the bulk substituents of NHC ligands lead to predominantly monodentate coordination of the pyrazoles. Therefore, the binuclear derivatives (for L = tfpz) in this case are formed via argentophilic contacts, rather than the interaction of pyrazolate fragments. The combination of bulk groups in pyrazolate (R = *^t^*Bu, R′ = C_3_F_7_) ensures the formation of monomeric complexes. A decrease in the nuclearity of the structures leads to a significant decrease in melting temperatures (~100 °C). However, according to sublimation tests, all adducts have similar volatility with the initial silver pyrazolates, which may be due to their comparable molecular weights. Due to the combination of synthetic availability and thermal properties, a binuclear adduct with Q = *^i^*^Pr^NHC (1,3-di-iso-propyl-imidazolin-2-ylidene) can be recommended for testing in deposition processes.

#### 3.3.3. Amide and Diiminate Complexes

**Figure 11:**
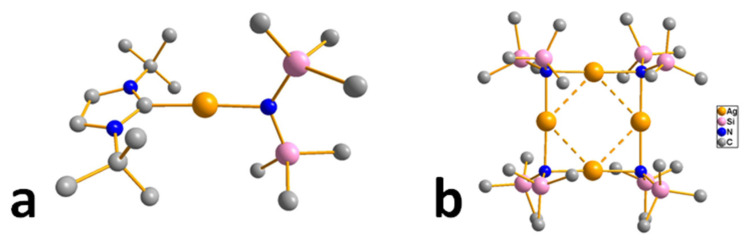
The molecular structures of silver complexes with silanaminide ligand: [Ag(*^tBu^*NHC)(hmds)] (**a**); [Ag(hmds)]_4_ (**b**). Hydrogen atoms were omitted for clarity. The cif-files were retrieved from CCDC. CCDC numbers are 1858520 and 1266662, respectively.

Although these ligands differ in their chemical structure, they can be combined by a common aspect. In the case of silver, complexes that can be used for MOCVD/ALD are represented by corresponding adducts with the same N-heterocyclic carbene (Q = *^tBu^*NHC, Figure 11a). The study of volatile carbene derivatives of silver was started with the adduct [Ag(*^tBu^*NHC)(L)] with 1,1,1-trimethyl-*N*-(trimethylsilyl)silanaminide (L = hmds) [41]. This complex was obtained with a yield of about 80% as a result of a direct reaction between AgCl, the corresponding imidazolium chloride, and the available lithium salt of aminosilane. The structure of this compound turned out to be mononuclear, as expected (Figure 11a). The vapor mass spectrum showed peaks of the molecular ion and its defragmentation products. The high thermal stability of this complex made it possible to use it not only in MOCVD but also in ALD experiments (see Section 4). It should be noted that homoleptic silver silanamide has a tetra-nuclear structure [Ag(hmds)]_4_ (Figure 11b) and is even more stable in the condensed phase (up to 275–280 °C compared to 225 °C for the adduct); however, information about its volatility was not presented [100].

On the other hand, [Ag(*^tBu^*NHC)(hmds)] is a suitable reagent for expanding the library of such carbene derivatives due to the ease of direct substitution of silamide. This made it possible to obtain the corresponding adduct with N-methyl substituted acetylacetone derivative (L = NacNac, pentane-2,4-methyiminate), for which no other silver complexes have been described [101]. According to NMR, in the adduct, this β-diiminate ligand exhibits chelate coordination typical of β-diketonate derivatives. The complex begins to decompose already at 45 °C, which opens up prospects for low-temperature deposition processes, provided that a system for introducing the precursor into the reactor is simplified.

#### 3.3.4. N-Succinimide Complexes

**Figure 12:**
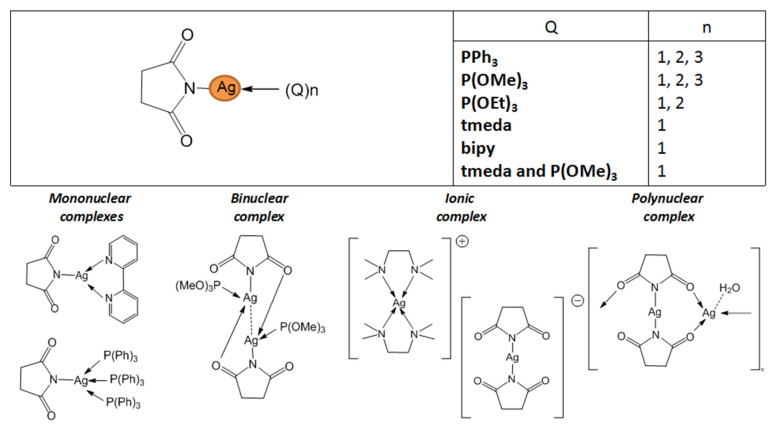
Silver N-succinimide complexes.

It should be noted that the classes of N-donor ligands discussed above are typical candidates for the design of volatile metal complexes. However, succinimide (NC_4_H_4_O_2_^−^, L = succ; Figure 12) was considered in this role only for silver. 

This is probably due to the fact that this combination has already found commercial use. Succinimide is used as an effective complexing agent in electrolytes for the silver electroplating process [102]. This may stimulate interest in expanding the applications of the complexes, including the search of succinimide-based silver precursors for MOCVD processes [103,104,105].

Due to the small size and rigid spatial organization of the ligand, silver succinimide is expected to have a polymeric structure, which has been shown for the corresponding aqua-derivative [106]. Although the blocks {Ag_2_(L)_2_} with stoichiometry similar to carboxylates and amidinates can be distinguished in the structure, in this case, their structure is asymmetric, and the Ag...Ag distance is significantly larger (>3.1 Å). Periodic quantum chemical calculations did not reveal argentophilic interactions in the crystal [107], whereas according to NMR data, the dissociation of the binuclear block into cationic Ag(Solv)^+^ and anionic Ag(L)_2_^−^ parts [108] proceeds in solution. Both observations indicate the absence of stabilization of {Ag_2_(L)_2_} blocks for L = succ, which could persist in the gas phase, similar to the case described for silver carboxylates and amidinates. Therefore, although detailed data on the thermal properties of silver succinimide at atmospheric/reduced pressure have not been found, it is assumed that a decomposition process takes place during heating rather than a transition to the gas phase.

Thus, in order to obtain potentially volatile succinimide derivatives, stabilization of complexes by P- and N-donors such as PPh_3_, P(OEt)_3_, P(OMe)_3_, and tmeda was considered [103,104,105]. These adducts can be obtained through the direct reaction of “Ag(succ)” with stabilizing ligands Q or by in situ assembly from AgNO_3_, Q, and Hsucc using triethylamine as a base [105]. The obtained compounds (Figure 12) are sensitive to light, oxygen, and humidity, probably with the exception of the adduct with bipy, for which special synthesis and storage conditions have not been noted [109]. In the case of phosphines and phosphites, the formation of adducts with the stoichiometry Q:Ag(L) = 1, 2, and 3 [103,104], depending on the ratio of reagents, is possible. Note that the derivatives with Q:Ag(L) = 2 and 3 for P(OEt)_3_ and P(OMe)_3_ are liquid.

According to single-crystal XRD, there are several types of organization of adduct structures (Figure 12) [103,104,105]. Thus, the introduction of a single monodentate ligand PR_3_ is apparently insufficient to stabilize the monodentate coordination of N-succinimide due to steric effects, and binuclear complexes [Ag(PR_3_)(L)]_2_ are formed. Unlike the initial succinimide, the environment of the metal centers in these complexes is already the same, and additional stabilization occurs due to the weak argentophilic contact. Further addition of the coordination sphere of silver leads to the formation of mononuclear adducts, except for the tmeda diamine derivative. In this case, separation into cationic Ag(tmeda)_2_^+^ and anionic Ag(L)_2_^-^ parts occurs. At the same time, for the aromatic analogue with Q = bipy, no such effect is observed [109]: the formation of a mononuclear complex with T-shaped {AgN_3_} coordination appears to be stabilized by π-stacking. The solution to the problem in the case of Q = tmeda could be the introduction of an additional monodentate neutral ligand, as demonstrated by the example of Q_1_ = P(OMe)_3_. This resulted in the formation of the [Ag(tmeda)(Q_1_)(succ)] adduct, which was liquid [105].

Almost all solid compounds melt with decomposition with melting temperatures ranging from 165 to 200 °C. The only exception is the low-melting adduct Ag(L)·P(OEt)_3_ (53–55 °C). In general, information about the thermal properties of these complexes is sporadic and insufficient for a comparative analysis. For instance, TGA data were found only for three compounds (Ag(L)·Q, Q = P(OEt)_3_, 2P(OMe)_3_, and 2PPh_3_). These data indicate the decomposition of the complexes when heated under atmospheric pressure. No information is available about sublimation under low pressure. Although liquid adducts with phosphites Ag(L)·2Q, Q = P(OEt)_3_, and P(OMe)_3_ have been successfully tested in the classic thermal MOCVD process [104,105], the resulting films have not been characterized in sufficient detail. Therefore, it is advisable to verify the results presented in these studies (see Section 4.1).

### 3.4. Other Complexes

Other classes of silver complexes have rarely been tested or even proposed for the application of gas-phase processes, and, as a rule, the relevant examples are limited to single works. In general, the use of monodentate anionic ligands is a rare design strategy for low-nuclear volatile silver complexes, since the low charge density of the cation complicates the selection of appropriate candidates with the necessary electronic and steric characteristics. Therefore, complexes with bidentate ligands, including chelating ligands, have been primarily considered (Table 2). 

#### 3.4.1. Complexes with O and (O^O) Donor Anionic Ligands

*Alkoxides.* Although these ligands are widely used for the design of volatile precursors [45], they act as effective reducing agents towards Ag^+^ [110], which explains the known instability of the corresponding derivatives [111]. Later, it was shown that special fluorinated ligands, in particular, bulky L^F^ = OC(CF_3_)_3_, can stabilize silver complexes [112]. The complex is prepared by a metathesis reaction between LiL^F^ and AgF or AgBF_4_ in an inert atmosphere with light protection. The compound has a one-dimensional polymer structure, in which tetra-nuclear building blocks {Ag_4_(L^F^)_4_} can be distinguished. Apparently, the presence of these “stabilized” fragments determines the ability of the complex to sublime at 130 °C and 0.0075 Torr. Vaporization in the form of similar tetra-nuclear molecules has also been observed for an organometallic silver derivative (see Section 3.5.3). Thus, (per)fluorinated silver alkoxides may be interesting for gas-phase processes, and recent achievements in synthetic approaches [113,114] can support the development of this area of chemistry, both for monoligand complexes and for stabilized adducts.

*Aryloxides.* An alternative way to stabilize silver salts with monodentate O donors is to use phenols, especially with electron-withdrawing groups [111]. Like alkyl oxides, monoligand aryloxides of silver are coordination polymers, but structural blocks {Ag_3_(L)_3_}, which are supported by contacts of silver with substituents (Cl- and NC-) and phenyl rings, can be distinguished in their structure [115,116]. Such a number of intermolecular interactions prevent vaporization. For this reason, it is advisable to consider adducts as potential volatile aryloxide precursors of silver.

The screening of such precursors was started with P-donors (Q = phosphines and phosphites). A set of 16 compounds for mono- and tri-substituted phenols (Table 2) was investigated in Refs. [115,117]. Adducts are easily synthesized either by direct reaction of the corresponding phenolates with neutral ligands, or through in situ assembly from Ag_2_O [115]. They are more stable when stored at room conditions compared to phenolates. For Q = 2P^n^Bu_3_, the corresponding derivatives are liquid, and information about the structure of crystal complexes is sketchy. The studied pair of mono-adducts regardless of the stabilizer size Q = P(OMe)_3_, P(OCH_2_CF_3_)_3_) has a tetra-nuclear structure with a typical Ag_4_O_4_ heterocubane core, where each metal center additionally coordinates a neutral ligand [117],whereas the studied *tris*-adduct (Q = PPh_3_) turned out to be mononuclear [115]. 

Thus, it can be assumed that the structure of adducts mainly depends on the Q/Ag ratio. However, more representative series for other types of L demonstrate the possibility of greater structural diversity (e.g., Section 3.1.2). According to TGA data, all adducts decompose in one or two steps at atmospheric pressure, and their thermal stability correlates with the expected electronic effects of the substituents. For example, decomposition temperatures for trisubstituted phenols (L = OPh^R3^) increase with the introduction of more acceptor groups: 55 °C (R = CH_2_N(CH_3_)_2_) << 195 °C (Cl) [115]. Thus, the most promising properties should be expected from nitro derivatives. Indeed, for a liquid adduct (Q = 2P^n^Bu_3_ and OPh^NO2^), the process of thermal vapor decomposition, which occurred at 190 °C, was studied by in situ mass spectrometry [117]. In general, the manifested volatility allowed for the use of this adduct in MOCVD with pulsed spray evaporation technique [117], whereas the derivatives of Q = PPh_3_ were ineffective even in the AA-MOCVD process [115].

*Sulfonates.* Derivatives of sulfonic acids, especially fluorinated ones (e.g., triflate ion L = OSO_2_CF_3_ = OTf), are classic sources of silver in coordination chemistry. It was shown back in the early 1980s that the Ag(OTf) complex transfers into the gas phase in the form of dimmers and is stable up to 380 °C; however, it is significantly less volatile compared to silver carboxylates [118]. A detailed spectral and thermal study confirmed these results and allowed researchers to explain them in terms of a more rigid structural organization: the complex is a layered coordination polymer in which each oxygen atom of the ligand performs a µ2 function [119]. Thus, only the development of the AA-MOCVD method made it possible to test Ag(OTf) as a commercial precursor [120]. Although a representative set of stable silver triflate adducts of molecular structure of various nuclearities, for example, with S- and P-donors [121,122,123], is described in the literature, we were unable to find data on their thermal properties to assess the possibility of increasing volatility.

At the same time, similar studies have been conducted for non-fluorinated sulfonic acid derivatives, namely silver methane(di)sulfonates (L = OSO_2_CH_3_ [124,125], or L = (OSO_2_)_2_CH_2_), which were not volatile [118]. Soft Lewis bases (Q = PPh_3_, P(OMe)_3_, P(OEt)_3_) were used as neutral ligands. As in previous cases, adducts are easily synthesized by reacting the corresponding silver salt with Q. The ratio Q/Ag = 1, 2, and 3 can be varied by changing the stoichiometry of the reaction mixture (Table 2) Note that the use of Q = phosphites makes it possible to obtain liquid complexes in the case when Q/Ag = 2,3, and even for a two-charge disulfonate–anionic ligand [118,124,125]. When Q/Ag = 1, disulfonate adduct has a chain polymer structure Q = P(OMe)_3_) [126], whereas in the case of sulfonates, tetra-nuclear complexes differing in structural organization are formed: with the Ag_4_O_4_ heterocubane core described above, including one µ3 oxygen atom of the anion (Q = PPh_3_) [124], or combined Ag_2_O_3_ cores with two anion coordination modes (Q = P(OMe)_3_) [125].

The related adduct with Q = 2PPh_3_ already has a multinuclear structure with bidentate sulfonate coordination [124]. In the sulfonate series, thermal properties have been studied only for a few compounds. For this reason, it is difficult which to identify any regularities other than the higher thermal stability of adducts with Q = PPh_3_ (melting with decomposition at 187–215 °C for Q/Ag = 1, 2, and 3) [124,125]. Disulfonates decompose under TGA conditions, and the mass loss curves of the adducts with Q/Ag = 2.3 are close. Both complexes are less thermally stable compared to the polymer analog (Q/Ag = 2.3): their decomposition onset is 90 °C vs. 120 °C [126]. Liquid derivatives with Q = 2P(OMe)_3_ were tested in MOCVD at deposition temperatures of 420 °C and 480 °C for L = OSO_2_CH_3_ and (OSO_2_)_2_CH_2_, respectively [125,126] (see point 4).

*N-hydroxysuccinimide.* Although N-hydroxysuccinimide (NHS, Table 2) is a widely used reagent in organic and bio-organic synthesis [127], its coordination chemistry has not been extensively studied. For example, a search of the Cambridge Structural Database reveals only five deposited structures with this ligand. The corresponding silver complex can be obtained with a high yield by the reaction of AgNO_3_ with NHS, deprotonated with a small excess of triethylamine [128]. However, the complex decomposes upon heating, likely due to its polymeric structure [129]. 

A series of adducts with conventional P-donors, viz. Q = PPh_3_, P(OMe)_3_, and P(OEt)_3_, have been studied as potential volatile precursors [128]. Similarly to the case described above for sulfonates or N-succinimides (L = succ), the synthesis was carried out through the direct reaction of the corresponding silver salt with Q. However, products were obtained for only two ratios, Q/Ag = 1 and 2 (Table 2). This appeared to be due to the chelated (O^O)-coordination of the NHS anion, which was confirmed using X-ray diffraction for the derivative with Q = 2PPh_3_ [128]. Following the general features, the introduction of phosphite ligands led to the formation of liquid adducts. High sensitivity of the compounds to moisture and oxygen, as well as light, was noted: despite the chelating ligand, these derivatives are apparently less stable than N-succinimide analogues [105]. Unfortunately, the data on thermal properties do not allow us to compare these related classes, because these data are sporadic and mainly presented for different Q. Nevertheless, it can be noted that the incongruent decomposition temperatures for Q = 2PPh_3_ are comparable (185 °C for NHS anion; 195 °C for L = succ), and the mass loss curves of analogues for Q = 2P(OMe)_3_ have a close character [104,128]. However, the deposition temperatures for MOCVD from N-hydroxysuccinimide adduct (Q = 2P(OMe)_3_) were significantly higher than for N-succinimide derivatives: 480 vs. 350 °C [104,105,128].

#### 3.4.2. Complexes with (N^O) Donor Anionic Ligands

*N-acetylbenzamides.* Although these ligands were proposed as structural analogues of β-diketones, in silver complexes, they showed (N^O) coordination, forming a less stable four-membered metallocycle similar to carboxylates (Figure 2) [130]. Although such coordination was directly shown for only one derivative, it was confirmed by the stoichiometry of the obtained adducts with P-donor ligands (Q = PPh_3_ and P(OEt)_3_, (Table 2)). In fact, a complete set of adducts was obtained for the ratios Q:Ag(L) = 1, 2, and 3, as well as for carboxylates. However, an increase in the metallocycle due to steric effects consistently reduces the number of coordinated P-donors: Q:Ag(L) = 1–2 for N-hydroxysuccinimides (*five-membered metallocycle*, see Section 3.4.1), Q:Ag(L) = 1 for β-diketonates (*six-membered metallocycle*, see Section 3.2.2). These adducts were synthesized using an in situ assembly reaction from AgNO_3_, Q, and N-acetylbenzamide sodium, varying the ratio of reagents.

Although these compounds are sensitive to air, they remain stable at room temperature in an inert atmosphere. In the case of Q = PPh_3_, the adducts melt with decomposition at temperatures above 205–222 ° C. For derivatives at Q:Ag(L) = 2 and 3, these values are close, whereas the analogue with Q:Ag(L) = 1 is slightly less thermally stable (the difference is ~10 °C). In the case of Q = P(OEt)_3_, all adducts are liquid. However, the thermal properties are given only for Q:Ag(L) = 2: according to TGA, decomposition begins at temperatures of ~100 °C. This adduct has also been tested in MOCVD. At a load of 500 mg and a temperature of 400 °C, individual spherical silver particles were obtained, while when the temperature was increased to 450 °C, the deposition of trace amounts of silver occurred. It should be noted that, in this case, the highest vaporization temperatures were set (120 °C vs. 70–80 °C) compared with other classes tested by the same scientific group (N-(hydroxy)succinimides and methane(di)sulfonates; see Section 3.3.4 and Section 3.4.1), which may indirectly indicate a lower volatility of N-acetylbenzamide derivatives.

*Oxyquinolinate*. Among the (O^N)-donor ligands forming five-membered metallocycles, 8-hydroxyquinoline derivatives were considered for the design of potentially volatile silver complexes (Table 2) [117]. These derivatives have shown efficacy in the case of Au(I) [131]. In particular, adducts with Q = P^n^Bu_3_ with the ratios Q:Ag(L) = 1 and 2, typical for five-membered chelating anionic ligands (see above). were synthesized according to the general approach described above for aryl oxides (Section 3.4.1). Unlike the corresponding silver quinolinate, these adducts are less demanding in regard to storage conditions and soluble in usual organic solvents [117]. According to the TGA results, the adduct with the stoichiometry Q:Ag(L) = 1 is thermally more stable compared to Q:Ag(L) = 2 (the beginning of decomposition is 130 °C vs. 100 °C). However, the results of its testing in deposition processes were not provided. It should be noted that silver easily forms molecular adducts of the composition Ag(HL)(L), which may have a mono- or binuclear structure [132,133]; however, information on the thermal properties of such derivatives has not been found.

*β-Ketoiminate*. Among (O^N) donor ligands forming six-membered metallocycles, β-ketoiminates (Table 2) represent the most promising compounds for the synthesis of volatile precursors. They are considered as analogues of β-diketonates, which have a similar chelation effect and internal conjugation, but at the same time open up additional possibilities for structural modification due to the substituent R′ at the nitrogen atom. In fact, they are proposed as a “reasonable compromise” between the stability and availability of β-diketonates and the reactivity of (N^N) coordinated ligands. In the case of silver, compounds of this class are represented by adducts with phosphines (Q = PPh_3_ and PBu_3_) [61,79] and N-heterocyclic carbene (Q = ^tBu^NHC) [101]. It is important to note that attempts to isolate monoligand non-fluorinated β-ketoiminates were unsuccessful (unlike β-diketonate analogues) [61]. Therefore, for each type of adduct, its own unique synthesis method has been developed.

In particular, non-fluorinated adducts with phosphines were obtained as a result of the reaction of AgNO_3_ with the sodium salt of β-ketoimine in the presence of a neutral ligand [61]. Fluorinated adducts were synthesized through nucleophilic addition of amines to the corresponding β-diketonate adducts [79]. All of these adducts decomposed upon heating. However, the fluorinated derivatives have shown promising results in AA-MOCVD deposition process, providing smoother films with fewer carbon impurities compared to β-diketonate analogues [79].

The unfluorinated adduct with carbene was synthesized by a standard reaction of hmds ligand substitution from the corresponding β-ketoimine derivative (see Section 3.2.2 and Section 3.3.3), but with a lower yield than β-diketonate and β-diiminate analogues [101]. According to NMR data, the complex exists in solution in the form of two structural isomers—with chelating and monodentate coordination of the anionic ligand—whereas the latter form is stabilized in the crystal, and coordination occurs through the N-center. Similar features did not appear for related complexes. As a result, the β-iminoketonate derivative has the lowest thermal stability and decomposes, as shown by TGA, with the maximum relative mass residue. In this regard, it is interesting to check whether chelate coordination can be achieved by changing the structure of the β-ketoiminate fragment.

**Figure 13:**
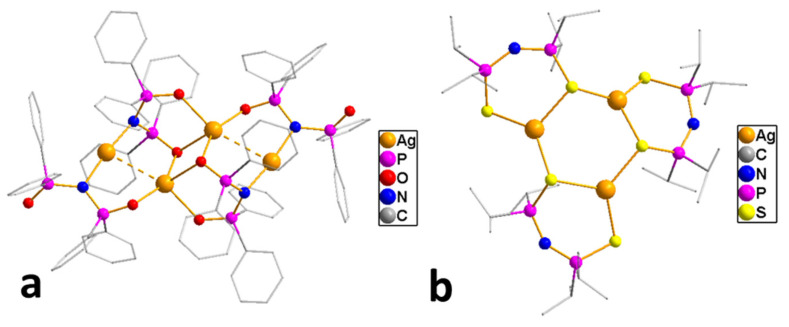
Silver precursors of imidodiphosphinate type: dioxoimidodiphosphinate [Ag{(OPPh_2_)_2_N}]_4_ (**a**); dithioimidodiphosphinate [Ag{(SP(^i^Pr)_2_)_2_)N}]_3_ (**b**). Hydrogen atoms were omitted; phenyl groups are transparent for clarity. The cif-files were retrieved from CCDC. CCDC numbers are 693641 and 666128, respectively.

*Dioxoimidodiphosphinate*. Ligands belonging to the class of oxidized imidodiphosphates (Table 2) are structural analogues of β-diketonates but with greater flexibility due to P–N–P backbones [134]. Another feature of these ligands is that the central nitrogen atom can also be involved in coordination, increasing the denticity of the ligand from (O^O) to (O^N^O). However, such ligands are usually stabilized by aromatic substituents at phosphorus atoms [134], thus limiting their use for the design of volatile complexes. The development of AA-MOCVD processes facilitated the testing of the corresponding silver derivative [38]. The complex with tetraphenyldioxoimidodiphosphinate (L = {(OPPh_2_)_2_N} = TPOIP, Table 2) was synthesized by the reaction of silver trifluoroacetate with the ligand of deprotonated sodium methoxide. Note that the use of more traditional AgNO_3_ was ineffective. Unlike most of the considered compounds, the complex is resistant to moisture, oxygen, and light. This is due to both the impossibility of proton transfer and its tetra-nuclear structure stabilized by bridging (O^N^O) or (O^N) coordination of ligands, as well as a pair of intramolecular argentophilic contacts (Figure 13a). The complex melts at 243–246 °C and maintains its stability up to temperatures not lower than 340 °C (TGA data). In this regard, the growth of films in the AA-MOCVD process was observed only at 375 °C, but even at higher deposition temperatures, the samples contained phosphorus impurities.

#### 3.4.3. Complexes with S-Donor Anionic Ligands

The use of complexes with S-donor anionic ligands, among which *dithiocarbamates* (L = S_2_CNRR′_2_) are most often considered [135], is complicated by the presence of relatively strong M-S bonds in their structure, which contributes to the formation of sulfides. In fact, metal films can be formed only on the most inert metals, such as gold [131] and platinum [136]. It was found in early studies that in the case of silver, the decomposition of dithiocarbamates in an inert atmosphere occurs with the formation of Ag_2_S, whereas metallic silver can form in air at the first stage but in a mixture with sulfide and sulfate [137]. In accordance with these features, active studies of silver dithiocarbamates, including their use in AA-MOCVD [138,139], were focused on their use as single-source precursors of sulfide materials [135] and, therefore, are beyond the scope of this review.

The first approach to obtaining metallic nanomaterials from precursors with S-donor ligands is to select special conditions for selective bond breaking. For silver, this concept was demonstrated using *monothiobenzoate* (Ag(SCOPh)) as an example [140]. Since the dissociation energy of the Ag−S bond is significantly lower than that of S−C (206.41 vs. 265.95 kJ/mol), gradual heating of the complex solution leads to the formation of Ag nanocrystals. At the same time, when using hot injection, enough energy is immediately released to form Ag_2_S. This promising approach has not yet been adapted to chemical gas-phase deposition processes, but the development of appropriate equipment may open up new horizons for its application.

An alternative approach is to design the complex in such a way as to expand the “temperature window” or provide other conditions for a favorable cleavage of the Ag–S bond. A successful example of approach is the use of the *dithioimidodiphosphinate* ligand (L = {(SP(^i^Pr)_2_)_2_)N}, (Table 2), which was proposed as a structural analogue of the β-diketonate [141]. The corresponding silver derivative was obtained by reaction of AgNO_3_ with the ligand using sodium methoxide as the base for deprotonation in the first stage. The complex had a trinuclear molecular structure, where one of the sulfur atoms of the chelating ligand performed a bridging function, forming the Ag_3_S_3_ core (Figure 13b). Note that in comparison with the O-donor related derivative (*dioxoimidodiphosphinate*, see Section 3.4.2), the complex under consideration has significant features both in synthesis and in structure. Due to the lower nuclearity and internal stabilization of the molecule, it has a lower melting point and less thermal stability (280 °C vs. 340 °C, TGA data). It is important that it was metallic silver that was a final product of its decomposition (~480 °C). Due to the oligonuclear structure, the complex was not volatile enough to be used in classical CVD; however, in AA-MOCVD, at high deposition temperatures (475–525 °C), a gradient zone of metal film formation was detected [141]. Although information on the impurity composition has not been provided by authors, and the issue of gradient control remains unresolved, this example illustrates the potential of a “chemical” approach to the precursor design.

Direct S-derivatives of β-diketonates, in particular *mercaptoenones* (Table 2), are also of interest. Several such non-fluorinated silver complexes with bulk substituents in ligands were synthesized by the direct reaction of AgNO_3_ with the sodium salt of mercaptoenones. The introduction of phosphines (Q = PPh_3_, PBu_3_) into the reaction mixture allowed researchers to obtain the corresponding mono-adducts [61]. It was established that such adducts had a binuclear structure in which anionic ligands perform a bridging function similar to that described for [Ag(cod)(hfac)]_2_ (Figure 5b; see Section 3.2.2). In accordance with the affinity of silver, Ag-O bonds are longer compared to Ag-S.

Despite this, all complexes decomposed under TGA conditions to form metallic silver. By analogy with the case described above for L = {(SP(^i^Pr)_2_)_2_)N}, this makes them promising for testing in AA-CVD. Other structural analogues can also be considered for this application, for example, *N-(thiophosphoryl)thiourea* silver complexes, which were studied by Crespo et al. [142]. In particular, discrete silver films were deposited from the PPh_3_ adduct with L = RC(S)NHP(S)(O^i^Pr)_2_ despite the presence of bulk R = C_10_H_7_NH_2_ (Table 2) in the AA-CVD process [143]. Obviously, such samples do not have sufficient quality to be used as conductive, optical, or other layers, but this approach may be of interest for obtaining isolated nanoparticles.

Finally, “fine-tuning of the precursor” is also possible using traditional anionic ligands. In particular, among a wide range of dithiocarbamate adducts Ag(PBu_3_)*_n_*(L) (n = 1, 2, and 3), when decomposing a derivative with allyl substituents (R = R′ = CH_2_CH=CH_2_) for n = 3, pure silver was unexpectedly obtained instead of the typical Ag_2_S or Ag/Ag_2_S mixture [144]. Thus, more detailed studies of the decomposition processes are required for the design of S-donor-based precursors for the deposition of metallic silver.

#### 3.4.4. Complexes with P-Donor Anionic Ligands

**Figure 14:**
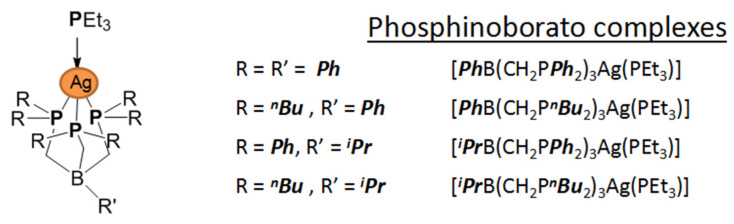
Silver tris(phosphino)borato complexes.

In the chemistry of silver precursors, P-donor neutral ligands are the most commonly used “stabilizers”. However, there are only a few examples of the use of P-donor anionic ligands in this area. In particular, a series of silver adducts with PEt_3_ for scorpionates of this type, i.e., tris(phosphino)borato ligands (L = RB(CH_2_PR′_2_)_3_, Figure 14), were considered [145]. 

These complexes were synthesized by the reaction of AgCl, PEt_3_ and [Li(tmeda)(L)] in an inert atmosphere, which was associated with special requirements for intermediate products, since the target complexes were resistant to air and light. In the case of R′ = iPr, the adducts were oily liquids. For solid adducts (R′ = Ph), the tridentate coordination of the scorpionate ligand, which ensures the formation of a stable mononuclear structure, was proved using XRD. The presence of a Ph substituent in the boron atom provides a stronger binding of the silver cation to the neutral ligand R due to better delocalization of the anionic charge. The corresponding adduct (R = R′ = Ph) is thermally more stable than the others (according to the TGA data, the difference in temperatures of the onset of mass loss is ~40–50 °C). However, such stabilization turned out to be “excessive”: the films obtained from it by the AA-CVD method contained a significant amount of boron, carbon and phosphorus impurities (in total, ~15 at.%, according to XPS). At the same time, the use of an analogue with R = ^n^Bu and R′= Ph allowed researchers to reduce the level of impurities to 5 at.%.

### 3.5. Organometallic Compounds

Although organometallic compounds, especially carbonyl or cyclopentadienyl derivatives, constitute a wide class of volatile precursors for many metals [146], in this case, their use is severely limited due to the specific features of silver chemistry.

#### 3.5.1. Carbonyl and Other Small Molecules Derivatives

The formation of carbonyls of coinage metals is difficult: the “classical” interaction includes both σ-donation of M ← CO and π-reverse donation of M → CO, whereas silver is a weak σ-acceptor, and donation is largely suppressed due to low-lying d-orbitals [147,148]. Thus, specialized anionic ligands combining steric effects and weak “soft” donor centers are used to stabilize Ag-CO adducts, among which scorpionate derivatives, such as tris(pyrazolyl)borates, L = [XB(R^F^Pz)_3_]^−^, where X = H, Me, Ph, R^F^ = 1, 2 or 3 perfluoroalkyl substituents [149,150]. 

Note that “stability” in this context usually means the absence of CO cleavage at room temperature in air and/or vacuum for several hours or days. Due to their high molecular weight, these derivatives are not expected to exhibit significant volatility, although some adducts may be thermally stable before melting. For instance, the most highly fluorinated compound [HB(3,4,5-(CF_3_)_3_Pz)_3_]Ag(CO) decomposes at 117–120 °C [150], while the methyl-substituted [MeB(3-(C_2_F_5_)Pz)_3_]Ag(CO) decomposes at 276–277 °C [151]. Therefore, such adducts are of theoretical interest for testing in AA-MOCVD and related processes.

Similar trends are observed for silver derivatives with ethylene or isocyanide [149,152]. The thermal stability of Ag-C_2_H_4_ adducts is generally comparable to that of Ag-CO analogues [147,148].

#### 3.5.2. Cyclopentadienyl Derivatives

Due to the certain geometry, cyclopentadienyl ligands are unable to saturate the coordination sphere of silver within the formation of molecular fragments. Thus, low-nuclear Cp-based silver complexes should contain stabilizing neutral Q ligands, among which efficiency and versatility are characteristic of P-donors (see Section 3.1.2 and Section 3.2.2, etc.). However, these adducts are extremely sensitive to oxygen, moisture, temperature, and light, which remains a major challenge. Even in the case of the most spatially shielding phosphine PPh_3_, the Ag(PPh_3_)(Cp) complex decomposes within a few minutes at room temperature already in an inert atmosphere [153] (it is synthesized and stored at −70–80 °C [154]).

Three main strategies of stabilization due to the Cp ligand were considered: (1) steric effects, (2) increase in denticity, and (3) decrease in electron density in the ring. The first approach turned out to be the least effective: the introduction of three SiMe_3_ substituents into the Cp ligand increased the decomposition temperature to 32 C (Q = PBu_3_, an X-ray study was conducted) [155], and the introduction of five Ph groups led to its increase only up to 40 °C (Q = PEt_3_) [156], while maintaining sensitivity to air.

The introduction of a PPh_2_ substituent led to the formation of an additional donor center, which coordinated to the second silver atom. These binuclear complexes, [Ag(PR_3_)(^PPh2^Cp)]_2_, had a symmetrical structure and were stable in air, withstanding heating up to 125–180 °C (depending on the R group) [156]. The third approach showed significant potential. The introduction of acceptor groups, such as CO_2_CH_3_ or C(O)CH_3_, ensured the stability of the complexes in air at temperatures up to 50 °C. Thermal stability could be further increased by replacing the P-donor with a bidentate analog [156]. Finally, the introduction of five CF_3_ groups into the Cp ring provided increased oxidative resistance, making the complexes stable when exposed to oxygen, moisture, temperature, and light (Q = P^t^Bu_3_) [153]. Of the latter complex, η^3^/η^1^-hapticity of the ligand should be noted.

Thus, in general, the combination of P-donor and electron acceptor-substituted Cp rings can be effective for creating silver complexes suitable for testing in MOCVD processes. Similarly to carbonyl derivatives (Section 3.5.1), the decomposition pathways of such derivatives, which may differ from those of the already tested complex compounds, could be advantageous.

#### 3.5.3. Alkenyl and Other Derivatives

**Figure 15:**
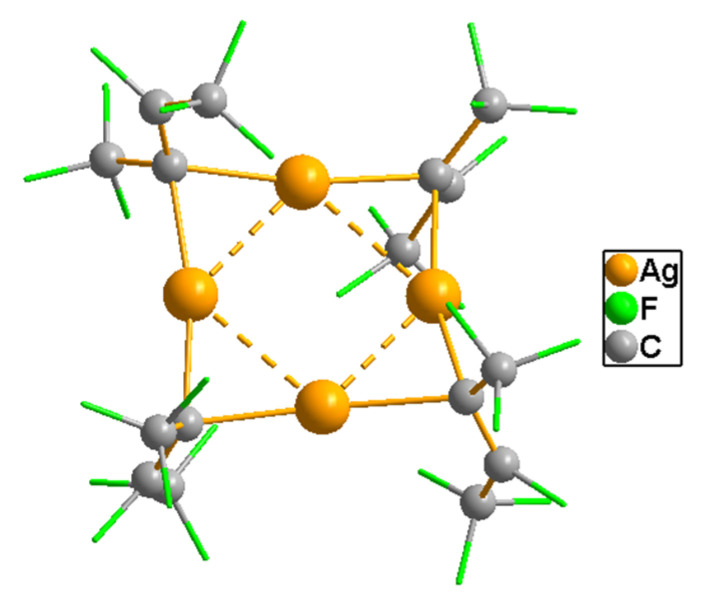
Silver organometallic precursor [Ag(C_4_F_7_)]_4_. The cif was retrieved from CCDC. CCDC number is 1202967.

Since Ag(I) belongs to highly oxidative metal centers [153], the stability problem associated with oxidative decomposition is common to organometallic silver compounds. Exceptional sensitivity to temperature, light, and humidity, which does not allow for the storage and use of σ-bonded organosilver complexes under standard conditions, may be less pronounced for some per- and polyfluoroorgano derivatives [157,158]. These include the only organometallic silver derivative tested in MOCVD processes, *trans*-perfluoro-1-methyl-1-propenylsilver [Ag(C_4_F_7_)]_4_ (Figure 15) [35,157]. The compound was obtained in 1969 as a result of nucleophilic addition of AgF to the corresponding alkyne (perfluoro2-butyne). Unlike the non-fluorinated analog, the complex decomposes at room temperature and is stable up to 175 °C [159]. The compound has attracted attention due to its unique (among its relatives) ability to sublime in vacuum, and later the existence of tetrameric particles in the gas phase was confirmed using mass spectrometry [35]. This is explained by its tetra-nuclear structure in which the metal centers are arranged in a square so that stabilizing argentophilic contacts can be realized (Figure 15) [157].

It has been shown that thermal decomposition proceeded through a “reverse” reaction, i.e., with the formation of AgF and perfluoro-2-butyne [157]. Taking into account the stability of AgF, this makes it possible to obtain sufficiently pure (<1 at.% of impurities according to XPS) films at 275 °C in thermal MOCVD (10^−4^ Torr) even in the absence of a reagent gas [157], whereas the introduction of Ar/H_2_ plasma reduces the deposition temperature to 120–150 °C while maintaining high film quality (resistance < 2 μΩ cm) [35].

However, despite these promising results demonstrated by different research groups, further development has not been pursued. Perhaps the development of methods for synthesizing organosilver derivatives [158,160] may reignite interest in this area and expand the range of potential precursors.

## 4. Ag Film and Nanoparticle Deposition

Table 3 and Table 4 show typical examples of the use of various classes of silver precursors in MOCVD and/or ALD processes. Some comparative features of these processes have already been discussed above (Section 3). A detailed discussion of the deposition processes of metallic silver may be the subject of a separate comprehensive review. In this review, we will briefly look at some general regularities and interesting results. 

In general, in classical CVD processes, adducts of organometallic compounds [Ag(Q)(L)]_n_, where L is β-diketonate (hfac, fod) or carboxylate (RCOO, R = C_2_F_5_, and C_3_F_7_) ligands, Q is neutral phosphine (PR′_3_, R′ = Me, Et, and Ph) or π-donor (cod, VTES) ligands, are most often used as precursors (Table 3). The choice of precursor and the process parameters influence the efficiency of obtaining pure silver nanomaterials.

**Figure 16:**
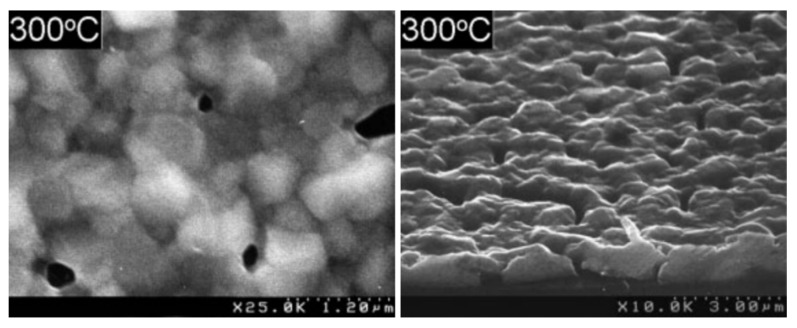
SEM image of an Ag film deposited from [Ag(tfpz)]_3_ in the presence of hydrogen. Reprinted with permission from [46]. Copyright 2005 John Wiley and Sons.

A few examples are given below. Despite the presence of fluorine in the composition of Ag hfac, fod adducts with phosphines, Ag films with low resistance were obtained in CVD experiments with various methods of the delivery of precursor vapors (Table 3). A comparative analysis of the effect of a neutral ligand on this functional characteristic showed that the best results were achieved in the case of π-donor VTES. This may be due to the lowest binding force to the metal center, which contributes to a more “clean” decomposition pathway [161]. However, too few samples have been studied so far to draw definitive conclusions. Film with resistance values close to the bulk material can also be obtained using precursors without additional ligands, as demonstrated using a pyrazolate complex [Ag(tfpz)]_3_ as an example. Ag films with a low resistance (3.6 µΩcm) close to the resistance of the bulk material (1.59 µΩcm) were obtained from this precursor in the presence of argon and hydrogen at 300 °C [46] (Figure 16).

**Table 3:** Deposition conditions and some characteristics of the Ag nanomaterials obtained by the variations in the MOCVD method.

Ref.	Precursor	Method *	Reagent	Source/Deposition (T, °C), Substrate, Particles Sizes (nm)	Composition (XRD) and Other Characteristics
[162]	[AgCOOC_2_F_5_]n	HW-MOCVD	-	240/280–350, titania nanotube coatings, 15–65	AgNPs, spherical crystallite shape
[163]	[Ag(cod)(hfac)]_2_	CW-MOCVD	H_2_	120/350, 5–20	AgNPs, spherical crystallite shape
**Ref.**	**Precursor**	**Method**	**Reagent**	**Source/Deposition (T, °C), Substrate, Thickness (nm), Growth Rate (nm/min)**	**Composition (XRD) and Other Characteristics**
[164]	[Ag(COOC_3_F_7_)]n	AA-MOCVD (THF)	-	160/310, glass, 90, 0.3–0.9	Ag, 4.2 μΩ·cm
	[Ag(PPh_3_)_2_(COOC_3_F_7_)]			160/310, glass, 65, 0.25–0.32	-
[79]	[Ag(PPh_3_)(hfac)]	AP-AACVD (THF)		-/310, glass, 306, 1.06	Ag, 186 μΩ·cm
	[Ag(PPh_3_)(hfacNhex)]			-/310, glass, 295, 1.64	Ag, 1.1 μΩ·cm
	[Ag(PPh_3_)(hfacNchex)]			-/310, glass, 248, 1.91	Ag, 167 μΩ·cm
[145]	[Ag(PEt_3_)PhB(CH_2_PPh_2_)_3_]	AA-MOCVD (THF)		-/300, glass or steel, 100, 0.5–0.6 (steel)	Ag > 85%, B-contamination, 2–3 µΩ cm
	[Ag(PEt_3_)^n^BuB(CH_2_PPh_2_)_3_]			-/500, glass or steel, 3000, 14–18 (steel)	Ag > 90%, B-contamination, 2–3 µΩ cm
[165]	[Ag(PEt_3_)(fod))]	DLI-MOCVD	N_2_/H_2_	40/250–300, SiO_2_/Si or TiN/Si, -	Ag = 59–99%, (F, Cl –surf. contamination), 3 μΩ cm
[9]	AgNO_3_	AA-MOCVD (alcohols)	-	-/147–427, TiN, glass, >900	>900 nm (2µΩ cm)
[161]	[Ag(cod)(hfac)]_2_	CW-MOCVD	-	60/200–250, TiN/Si, 500–720(only in case of [Ag(VTE)(hfac)])	Ag >99%, 1.92 μΩ cm
	[Ag(VTES)(hfac)]				-, 1.83 μΩ cm
[93]	[Ag(amd)]_4_		-/N_2_/H_2_	160–180/200–230, Si/SiO_2,_ 66–67, 0.26	Ag > 93% (N_2_/H_2_), 19.2–220 μΩ·cm
[94]	[Ag(pyrrolidinato)]_2_	HW-MOCVD	-	110/140–220, SiO_2,_ 5–156, 0.8–1.1	Ag > 96.8%, 2.46 μΩ·cm
[46]	[Ag(tfpz)]_3_	CW-MOCVD	-/H_2_	140/250–350, Si, 310–512, 5–8	Ag = 56–94%, >3.6 µΩ cm
[141]	[Ag_3_{(SP^i^Pr_2_)_6_N_3_}]	AA-MOCVD (Toluene)	-	-/425–525, glass	Ag + Ag_2_S (at 425–450 °C), Ag(111) (at 475–525 °C)

* HW-MOCVD—hot-wall chemical vapor deposition; CW-MOCVD—cold-wall chemical vapor deposition; AA-MOCVD—aerosol-assisted chemical vapor deposition; AP-AACVD—atmospheric-pressure aerosol-assisted chemical vapor deposition; DLI-MOCVD—direct liquid injection–chemical vapor deposition.

**Table 4:** Deposition conditions and some characteristics of the Ag nanomaterials obtained by variations in the ALD method.

Ref.	Precursor	Method *	Reagent	Source/Deposition (T, °C), Substrate, Particles Sizes (nm), (Number of Cycles)	Composition (XRD) and Other Characteristics
[40]	[Ag(cod)(hfac)]_2_	LI-ALD	Propanol	50/110–150, Si or SiN, 1–10 (110 °C, 1000 cycles) 22–60 (150 °C, 1000 cycles)	Ag, spherical or truncated octahedral crystallite shape, narrow size distribution Wulff model (110 °C)
[7]				130/123–128, Si(100), 10 (100 cycles), 25 (500 cycles), 48 (1000 cycles), 117 (1500 cycles)	Ag, spherical or oval crystallite shape, narrow size distribution, 0.17 Å/cycle
[166]			Tertiary butyl hydrazine	130/105–128, Si(100), 9 (500 cycles), 18 (1000 cycles), 27 (1500 cycles)	Ag, spherical or oval crystallite shape, 0.18 Å/cycle (thin films), 0.62–10^−5^ Ω cm
[39]	[Ag(PMe_3_)(hfac)]	T-ALD	HCHO or TMA+H_2_O	63–66/170–200, Al_2_O_3_, 2–19 or 1.8–2.2	Ag, spherical, narrow size distribution
[16]	[Ag(PEt_3_)(fod)]	PE-ALD (100 W)	H_2_	100/130–140, Si, 4 (100 cycles)	Ag, spherical or oval crystallite shape, Ag content 50–75%,0.2 Å/cycle
[167]		PE-ALD (100 W)		106/120, Ti, 6.8–9.6 (50 cycles)	Ag_2_O (no more than 10% XPS), spherical or oval crystallite shape, medical application
[168]		T-ALD	Me_2_NHBH_3_	96/100–120, Si, the filter of N95 mask, 16	Ag, spherical or oval, medical application
** Ref. **	** Precursor **	** Method **	** Reagent **	** Source/Deposition (T, °C), Substrate, Thickness (nm), Growth Rate (nm/min) (Number of Cycles) **	** Composition (XRD) and Other Characteristics **
[8]	[Ag(PEt_3_)(piv)]	RE-ALD	H_2_or H	125/140–160, Si trenches, 40, 1.2 (100 cycles)	Ag > 70%, 6 μΩ·cm
	[Ag(PBu_3_)(piv)]			125/140–160, Si trenches	No deposition
[169]	[Ag(PEt_3_)(fod)]	APS-ALD	H_2_/N_2_	-/120, rotation speed of 40 rpm, 80 (on Mo)	Ag, Volmer–Weber island growth, 3.5–10 μΩ cm
[41]	[Ag(^tBu^NHC)(hmds)]	APP-ALD	Ar+H_2_	100/120, Si or glass, 0.14 (900–1800 cycle)	Ag > 99%, 0.9 Ω/sq (resistivity: 10^−5^ Ωcm)

* T-ALD—thermal atomic layer deposition; LI-ALD—liquid-injection atomic layer deposition; PE-ALD—plasma-enhanced atomic layer deposition; APS-ALD—atmospheric-plasma spatial atomic layer deposition; RE-ALD—radical enhanced atomic layer deposition; APP-ALD—atmospheric=pressure plasma-enhanced spatial atomic layer deposition.

**Figure 17:**
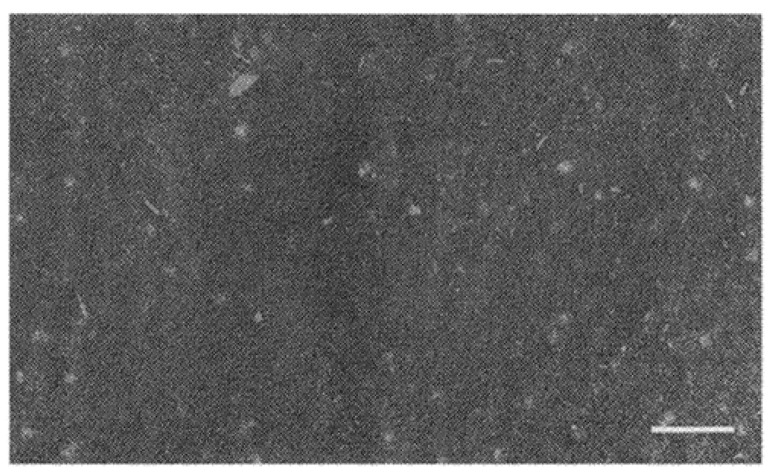
Scanning electron micrograph at 15 kV of a silver film obtained from the AACVD of Ag(C_3_F_7_CO_2_), bar = 10 μm. Reprinted with permission from [164]. Copyright 2002 Elsevier.

It was also found during the study of a series of phosphine adducts of Ag carboxylates that the use of fluorinated complexes led to the growth of more uniform reflective Ag films (Figure 17) in comparison with films obtained from non-fluorinated analogues.

**Figure 18:**
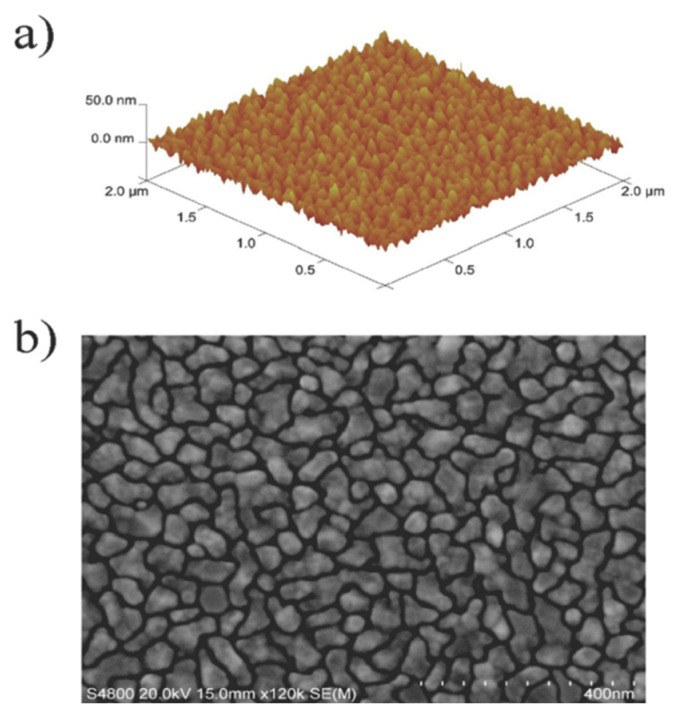
(**a**) AFM image of a 17 nm Ag film deposited at 120 °C from [Ag(PEt_3_)(fod)]. (**b**) SEM image of the same film. Reprinted with permission from [42]. Copyright 2011 American Chemical Society.

The development of the ALD method was accompanied by a narrowing of the range of silver precursors, for which high reactivity is important. [Ag(PEt_3_)(fod)], [Ag(PMe_3_)(hfac)], [Ag(cod)(hfac)]_2_ and [Ag(Q)(piv)] (Q = PEt_3_, PBu_3_) complexes, which contain an electron-accepting fluorinated (hfac) or non-fluorinated (piv) anionic ligand, as well as a neutral electron-donating phosphine (PMe_3_, PEt_3_) or an olefin (cod) stabilizing ligand, are mainly used in these methods (Table 4). The most commonly used is [Ag(PEt)_3_(fod)], which was tested in ALD processes in combination with various co-reagents, such as hydrogen, Me_2_NHBH_3_, or hydrogen plasma. In particular, thin films with a relatively small amount of impurities and a resistivity of 6–8 µΩcm were obtained from this precursor in the PE-ALD process with hydrogen (Figure 18, Table 4).

Next, some factors affecting the deposition processes of Ag-containing materials will be considered, including both experimental parameters and new promising precursors.

### 4.1. Application of Ag Precursors in MOCVD Processes

**Figure 19:**
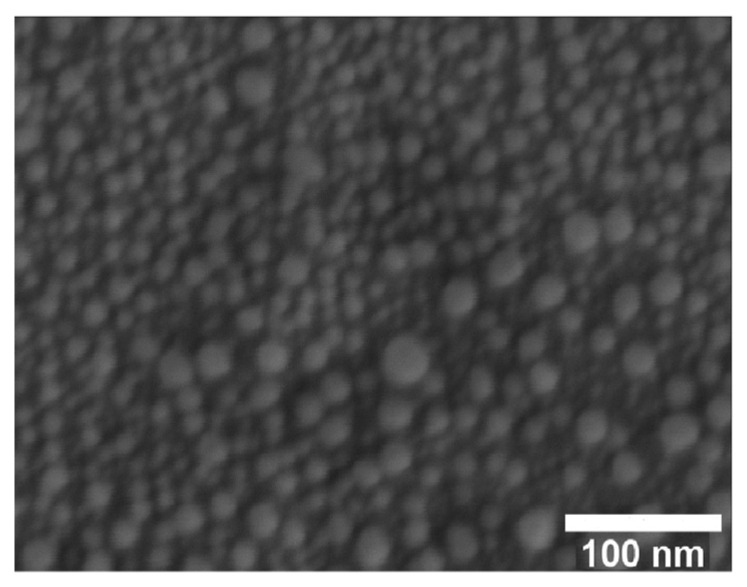
SEM image of Ag nanoparticles deposited from [Ag(cod)(hfac)]_2_. Reprinted with permission from [163]. Copyright 2024 MDPI.

Selection of experimental parameters. Since silver precursors have relatively low thermal stability and volatility, proper organization of vaporization processes can significantly increase the efficiency of experiments. This can be considered using the precursor [Ag(cod)(hfac)]_2_ as an example. Firstly, for this precursor, when using vaporization conditions suitable for the liquid and more volatile analogue, Ag(VTES)(hfac), (carrier gas flow rate of 3 l/h), no precipitated material was formed [161]. At the same time, an increase in the flow of carrier gas to 9 l/h in special evaporators made it possible to obtain both metal films [170] and nanoparticles [163] (Figure 19).

Secondly, an increase in the flow of reagent gas (hydrogen) to 12–60 l/h in both cases contributed to the achievement of high purity of the deposited materials.

**Figure 20:**
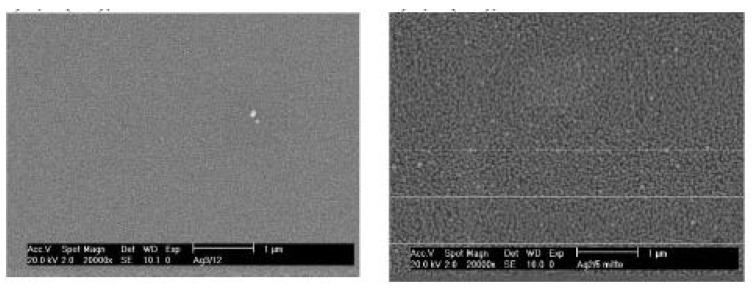
SEM images of silver films deposited from [Ag(PMe_3_)(piv)] (**left**), and [Ag(PEt_3_)(piv)] (**right**), on Si(100) substrates (cold-wall CVD, 190 °C). Reprinted with permission from [171]. Copyright 2005 Wiley.

The effect of the reagent gas. The tendency to improve the purity of coatings when hydrogen is introduced into the system (in mixtures or directly) seems to be common to the processes of the traditional flow MOCVD process. This can be explained both by the reducing effect, which prevents the formation of oxidized particles, and by the suppression of thermally induced ligand fragmentation. The low efficiency of the inert atmosphere and the introduction of hydrogen have been shown for different classes of silver precursors. For example, the replacement of argon carrier gas with hydrogen significantly reduced the level of impurities in films obtained from homoligand pyrazolates (56–84 at.% vs. 77–94 at.% silver) (Figure 16) [46]. In the absence of a reagent gas, films formed from non-fluorinated carboxylate adducts with phosphines (Q = PEt_3_ and PMe_3_) contained only up to 55 at.% silver, and the main impurities were oxygen and carbon (Figure 20) [171]. 

**Figure 21:**
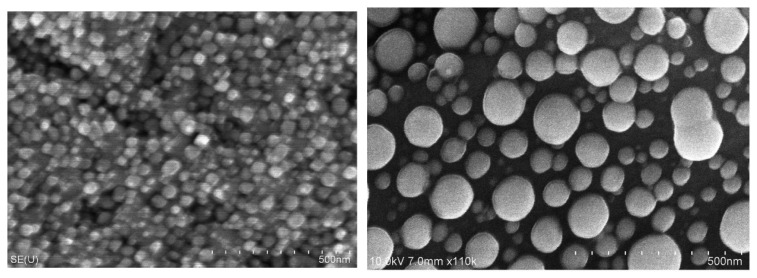
SEM images of silver films/nanoparticles deposited from N-succinimide [Ag(P(OEt)_3_)_2_(succ)] without a reducing agent at 350 °C (**left**) (reprinted with permission from [105]; Copyright 2011 John Wiley and Sons) and N-acetylbenzamide [Ag(P(OEt)_3_)_2_(NC_9_H_8_O_2_)] without a reducing agent at 400 °C (reprinted with permission from [130]; Copyright 2012 John Wiley and Sons) (**right**).

In this regard, a number of works describing the deposition of pure silver films without the use of a reagent gas for phosphine and phosphite adducts of such various classes as triflates, N-succinimides, N-hydroxysuccinimides, and N-acetylbenzamides seem illogical [104,105,128,172] (Figure 21). 

The reasons for this result, as well as the similarity of the morphology of the coatings in some cases, regardless of the precursor used, remain unclear and require additional study.

*Aerosol-assisted processes*. The development of methods for the deposition of silver from precursors with low thermal stability has become an effective approach, as evidenced by the growing number of studies in this area as well as the active development of chemistry of silver complexes. The variety of classes of such complexes is shown in Section 3 and Table 3. Moreover, a number of commercial silver precursors have been tested, including Ag(OTf) [120] and AgNO_3_ [9] (Table 3). However, negative results were obtained for a series of aryl oxides [117], adducts of *N-(thiophosphoryl)thiourea* [143], and some β-diketonate complexes [36]. One of the ways to initially assess the effectiveness of a precursor is to compare the value of the mass residue in TGA in an inert atmosphere with the calculated content of metallic silver. However, specific selection criteria, such as acceptable differences depending on temperature, have not yet been determined. Another important aspect is understanding the role of the solvent in the processes of precursor decomposition and film growth [9].

*Improvement of thermal properties in MOCVD.* Along with the development of precursor chemistry and variations in the MOCVD method, special techniques can be used to improve the thermal properties of already known compounds. One such approach is the use of mixtures of precursors or co-crystallizers, which can have a synergistic or association effect. For example, an increase in the thermal stability of silver acetylacetonate in a mixture with platinum acetylacetonate was shown: the difference mass loss temperatures in TGA was ~100 °C for Ag(acac) and the corresponding equimolar mixture [173]. A similar approach can be implemented in evaporators of “traditional” CVD installations. Another way to improve thermal properties is the formation of new or stabilization of existing complexes during in situ gas transport. For example, the formation of a bimetallic derivative was shown during the combined vaporization of copper and silver carboxylates [174]. In this way, it is possible to obtain mixed Ag-containing nanomaterials, which also have many applications.

### 4.2. Application of Ag Precursors in ALD Processes

Due to the strict requirements for the complexes (see Section 2), only a limited set of precursors was found to be suitable for ALD, namely phosphine- and cod-adducts of β-diketonates or carboxylates (Table 4). 

*Surface chemistry.* Ag ALD processes are difficult to study in surface reactions, since the film growth involves several mechanisms, and it is difficult to determine which of them is the predominant one [13]. Nevertheless, the use of quantum chemical calculations and in situ studies using Quartz Crystal Microbalance (QCM) allows researchers to identify some important features.

**Figure 22:**
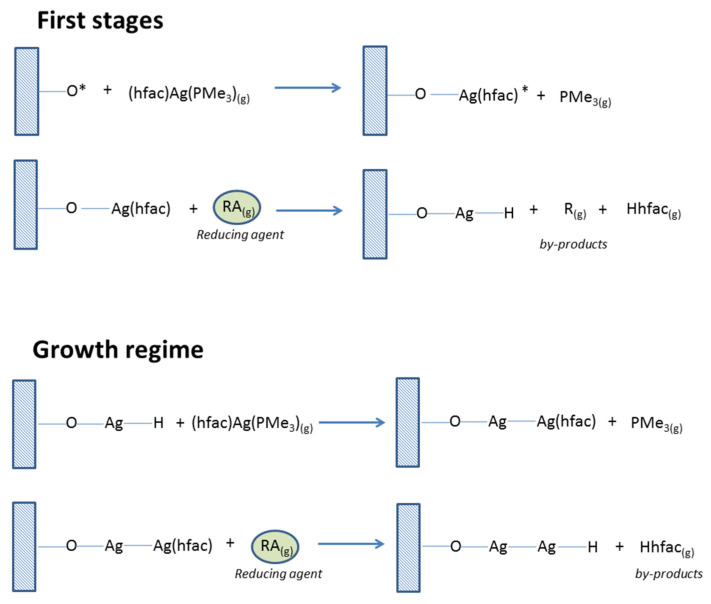
Proposed scheme of surface chemical reactions during the ALD process from silver β-diketonate adducts. The initial surface species are marked with an asterisk.

In particular, using Ag(PMe_3_)(hfac) as an example, it was theoretically proved that hydrogen bonds involving oxygen atoms of the substrate played a key role in the early stages of precursor chemisorption. This was experimentally confirmed by the fact that nanoparticles were not formed on the reduced surface of H–Si(100), unlike the surfaces of HO–Si(100) and silica [175]. Although we can follow the ideas about the stability of silver bond with the P- and O-donor ligand, further cleavage of the β-diketonate fragment rather than phosphine should be expected [175]. QCM studies showed the opposite order of destruction of the precursor (Figure 22) [39]. In the case of a more weakly bound neutral cod ligand, its dissociation can occur already in the gas phase, and the directly chemisorbed particle will be “Ag(hfac)” [7]. Further interaction with the reducing agent is assumed to release free β-diketone Hhfac as a result of catalytic dehydrogenation and to be accompanied by a “mild” oxidation of the co-reagent, i.e., alcohol to aldehyde, and formalin to carbon monoxide (Figure 22) [7,39].

In the case of carboxylate derivatives of Ag(Q)(piv), a strong dependence of the growth characteristics and properties of films on the neutral ligand was noted [8]. In the case of Q = PBu_3_, the growth rate decreased, while the impurity content increased, which may be the result of slower reaction kinetics or less efficient decomposition of enlarged alkyl fragments. This observation may also indicate the “reverse” order of ligand cleavage relative to the above described, when -Ag-PR_3_ is chemisorbed on the surface, which, in general, corresponds to the peculiarities of the thermal decomposition of these carboxylate adducts (see Section 3.1.1). However, the mechanism of film growth for such precursors has not been investigated. Thus, it is necessary to deepen the understanding of the surface chemistry of Ag ALD precursors in order to purposefully design new precursors.

*New precursors.* The N-heterocyclic carbene-based silver amide precursor (NHC)Ag (hmds) was later proposed as an alternative to O-donor ligand complexes (see Section 3.3.3) [41]. This adduct demonstrated approximately 2.5 times higher film growth rates than Ag(PEt_3_)(fod), and the films were also characterized by high purity [176]. However, no clearly defined “temperature window” was revealed, and saturation of surface reactions was not clearly proven, unlike with “traditional” ALD precursors [8,39]. This indicates the need for more detailed research in this area. One of the fresh ideas was the testing of silver amidinates in ALD processes, and more recently, theoretical studies have been initiated to evaluate the possibilities of chemisorption [177].

## 5. Conclusions

Thus, using CVD and ALD methods, it is possible to obtain innovative Ag-containing nanomaterials with precise control of the composition and other characteristics of Ag nanoparticles and film materials. The development of these processes is largely related to the search for new Ag CVD and ALD precursors. An analysis of literary sources indicated that significant progress has not yet been achieved in this area. A universal precursor has not been found. To obtain materials with the required characteristics, it is necessary to select the appropriate method and precursor. The development of methods for the synthesis of Ag complexes that will ensure the controlled growth of nanoparticles/thin films at low temperatures with a minimal level of impurities is still a significant challenge. However, recent studies have demonstrated that the use of a combination of certain ligands (e.g., based on carbenes) could expand the range of effective Ag precursors. Thus, the design and synthesis of heteroligand complexes of silver that could possess the necessary set of properties continue to progress actively.

## Figures and Tables

**Table 1:** The main types of silver β-diketonates adducts [Ag(Q)(L)]_n_.

**Adducts with π-donor ligands**					
*Q = cyclic π-donors*		*L = β-diketonates*	*Q = acyclic π-donors*		*L = β-diketonates*
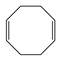	cod	hfac, tfac, ofhac	diphenylacetylene		hfac
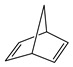	nbd	hfac	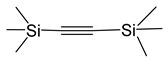	BTMSA	hfac, ttfac, btfac, fod
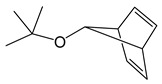	7-Bu’-O-nbd	hfac, ttfac, btfac, fod, tfac	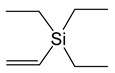	VTES	hfac, fod, tfac, ofhac
**Adducts with P-donor ligands**					
*Q = phosphines*		*L = β-diketonates*	*Q = phosphites*		*L = β-diketonates*
PMe_3_, PEt_3_		hfac, fod	P(OMe)_3_		hfac, tfac, efac
PBu_3_		thd, tmod	P(OEt)_3,_		hfac, tfac
PPh_3_		hfac, tfac, fod *, thd, tmod, acac	P(O^i^Pr)_3_		hfac, tfac
**Adducts with S-donor or with O-donor ligands**			**Adducts with carbene ligands**		
*Q = S-donors or O-donors*		*L = β-diketonates*	*Q = Carbene*		*L = β-diketonates*
**1,4-oxathian**e, SMe_2_, SEt_2_, S^n^Pr_2_, S^n^Bu_2_		hfac	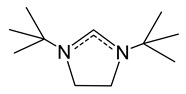	^t^BuNHC	hfac, fod, acac, dmm
CH_3_O(CH_2_CH_2_O)_m_CH_3_ (m =2–4)					
**Adducts with N-donor ligands**					
*Q = aromatic N-donors*		*L = β-diketonates*	*Q = aliphatic N-donors*		*L = β-diketonates*
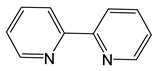	bipy	hfac, tfac, hfbac	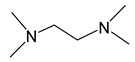	tmeda	hfac
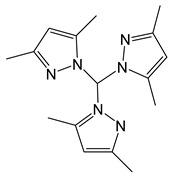	HC(3,5-Me_2_pz)_3_	hfac, tack	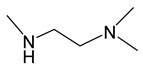	trimen	
			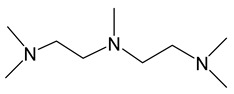	pmdien	
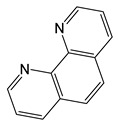	phen	hfac	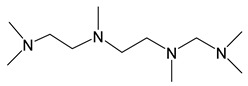	hmten	

* The stoichiometry is [Ag_3_(Q)_5_(L)_3_]_n_.

**Table 2:** The main types of silver complexes with O, S, P, and N donor anionic ligands.

**Monosubstituted aryloxide anion**				**Neutral ligand**				**Trisubstituted aryloxide anion**		**Neutral ligand**
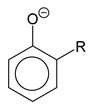		R = H		3PPh_3_ (phenol co-crystallizate)				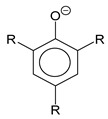	R = Cl	2PPh_3_
		R = Me		2PPh_3_ (o-cresol co-crystallizate)					R = (CH_3_)_2_NCH_2_	
		R = CHO or Cl		P^n^Bu_3_, 2P^n^Bu						
		R = CN		P^n^Bu_3_, 2P^n^Bu, P(OMe)_3_						
		R = NO_2_		P^n^Bu_3_, 2P^n^Bu, P(OMe)_3_, 2P(OMe)_3,_ P(OCH_2_CF_3_)_3_						
**Sulfonate complexes**										
Sulfonato anion *		**Sulfonate anion**		**neutral ligand**				**Disulfonate anion**	**Neutral ligand**	
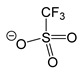		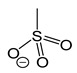		PPh_3,_ P(OMe)_3,_ P(OEt)_3_ 2PPh_3,_ 2P(OMe)_3,_ 2P(OEt)_3_ 3PPh_3,_ 3P(OEt)_3_				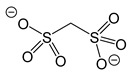	P(OMe)_3_, 2P(OMe)_3_, 3P(OMe)_3_	
**N-hydroxysuccinimide complexes**				**N-acetylbenzamide complexes**				**Oxyquinolinate complexes**		
*Anion*		*Neutral ligand*		*Anion*		*Neutral ligand*		*Anion*	*Neutral ligand*	
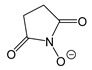		**PPh_3,_ 2PPh_3,_ P(OMe)_3,_ 2P(OMe)_3,_ P(OEt)_3,_ 2P(OEt)_3_**		** 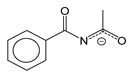 **		**PPh_3,_ P(OEt)_3,_ 2P(OEt)_3,_ 3P(OEt)_3,_**		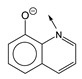	**P(OMe)_3_, 2P(OMe)_3_,** **3P(OMe)_3_**	
**β-Ketoiminate complexes**								**Dioxoimidodiphosphinate complex**		
*Anion*				*Anion abbreviation*		*Neutral ligand*		Anion *		*Abbreviation*
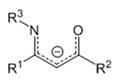		R^1^= R^2^ = CF_3_, R^3^ = R-hexyl or cyclohexyl R^1^= R^2^ = ^t^Bu, R^3^ = H R^1^= R^2^ = R^3^ = Me		**hfacNhex** or **hfacNchex** **Nthd** **NacacMe**		**PPh_3_**		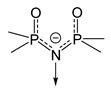		**TROIP**
						**PPh_3,_ P^n^Bu**				
						**PPh_3,_ P^n^Bu,** **^t^BuNHC**				
**Ditiocarbamates complexes**				**β-mercaptoenones complexes**				Dithioimidodiphosphinate complex *	**N-(thiophosphoryl)thiourea**	
*Anion*			*Neutral ligand*	*Anion*			*Neutral ligand*		*Anion*	*Neutral ligand*
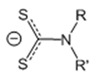	R = R′ = CH_2_=CH-CH_2_		**PPh_3_**	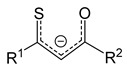	R^1^ = R^2^ = ^t^Bu		**PPh_3,_ PBu_3_**	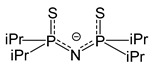	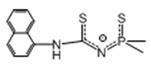	**PPh_3_**
					R^1^ = ^t^Bu, R^2^ = ^i^Bu					

* Complexes without neutral ligands.

## Data Availability

Not applicable.
